# Hypercapnia alters stroma-derived Wnt production to limit **β**-catenin signaling and proliferation in AT2 cells

**DOI:** 10.1172/jci.insight.159331

**Published:** 2023-02-22

**Authors:** Laura A. Dada, Lynn C. Welch, Natalia D. Magnani, Ziyou Ren, Hyebin Han, Patricia L. Brazee, Diego Celli, Annette S. Flozak, Anthea Weng, Mariana Maciel Herrerias, Vitalii Kryvenko, István Vadász, Constance E. Runyan, Hiam Abdala-Valencia, Masahiko Shigemura, S. Marina Casalino-Matsuda, Alexander V. Misharin, G.R. Scott Budinger, Cara J. Gottardi, Jacob I. Sznajder

**Affiliations:** 1 Pulmonary and Critical Care Medicine, Northwestern Feinberg School of Medicine, Chicago, Illinois, USA.; 2 Justus Liebig University, Universities of Giessen and Marburg Lung Center, Member of the German Center for Lung Research, Department of Internal Medicine, Giessen, Germany.; 3 The Cardio-Pulmonary Institute, Giessen, Germany.

**Keywords:** Cell Biology, Pulmonology, Respiration

## Abstract

Persistent symptoms and radiographic abnormalities suggestive of failed lung repair are among the most common symptoms in patients with COVID-19 after hospital discharge. In mechanically ventilated patients with acute respiratory distress syndrome (ARDS) secondary to SARS-CoV-2 pneumonia, low tidal volumes to reduce ventilator-induced lung injury necessarily elevate blood CO_2_ levels, often leading to hypercapnia. The role of hypercapnia on lung repair after injury is not completely understood. Here — using a mouse model of hypercapnia exposure, cell lineage tracing, spatial transcriptomics, and 3D cultures — we show that hypercapnia limits β-catenin signaling in alveolar type II (AT2) cells, leading to their reduced proliferative capacity. Hypercapnia alters expression of major Wnts in PDGFRα^+^ fibroblasts from those maintaining AT2 progenitor activity toward those that antagonize β-catenin signaling, thereby limiting progenitor function. Constitutive activation of β-catenin signaling in AT2 cells or treatment of organoid cultures with recombinant WNT3A protein bypasses the inhibitory effects of hypercapnia. Inhibition of AT2 proliferation in patients with hypercapnia may contribute to impaired lung repair after injury, preventing sealing of the epithelial barrier and increasing lung flooding, ventilator dependency, and mortality.

## Graphical abstract

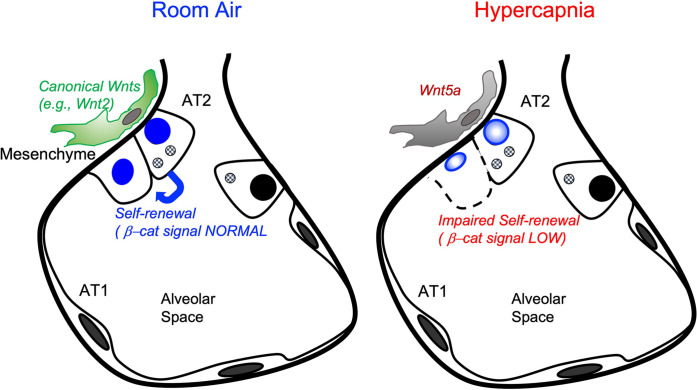

## Introduction

Severe COVID-19 presents as acute respiratory distress syndrome (ARDS) where injury of the alveolar epithelial barrier causes flooding of the alveolar space and pulmonary edema, which, in severe cases, may require mechanical ventilation (1). As of this writing, more than 651 million people have been diagnosed COVID-19 and nearly 6.7 million people have died (2). In the United States alone, there are currently more than 90 million survivors of COVID-19. Studies focused on postacute sequelae of COVID-19 (PASC) suggest persistent respiratory symptoms, where radiographic abnormalities and the need for supplemental oxygen are common in survivors of COVID-19, particularly those requiring high-flow oxygen therapy or mechanical ventilation (3). These persistent symptoms suggest that failure of normal lung-repair mechanisms can prevent complete recovery of lung function in a substantial fraction of COVID-19 survivors, with significant public health impact.

Low tidal volume ventilation is a proven strategy to reduce the incidence and severity of ventilator-induced lung injury (4–6). The alveolar hypoventilation associated with low tidal volumes necessarily increases blood CO_2_ levels; this elevation is described clinically as hypercapnia. Hypercapnia is exacerbated by increased steady state carbon dioxide production and dead space ventilation common in patients with severe SARS-CoV-2 pneumonia and ARDS (4, 6, 7). We and others found that exposure to hypercapnia activates signaling pathways detrimental to alveolar epithelial wound healing and migration (4–10), but mechanisms by which hypercapnia impairs epithelial repair remain incompletely understood.

While baseline turnover in the alveolar epithelium is slow, alveolar injury results in rapid and robust alveolar type II (AT2) cell proliferation and differentiation to restore barrier function and gas exchange (11–13). During homeostasis and after lung injury, lineage tracing studies suggest that AT2 cells self-renew and serve as progenitor cells for AT1 cells (13–16). Our group was among the first to report that Wnt/β-catenin (βcat) signaling regulates both the survival and migration of AT2 cells after injury (17). Recent studies suggest that a subset of AT2 cells with activated Wnt/βcat signaling display higher progenitor activity than the Wnt-inactive bulk population of AT2 cells (11, 16, 18, 19). Such differences in AT2 progenitor subsets are thought to be spatially induced in response to signals from the niche. For example, single-cell RNA-Seq data reveal that PDGFRα fibroblasts express Wnts, suggesting they might control the local activation of βcat AT2 cells (19–21), but the precise molecular signals and stromal cells that select AT2 progenitors are still undefined (16, 19, 21). Furthermore, whether and how signals responsible for maintenance and repair of the AT2/mesenchymal niche are affected by hypercapnia in the injured lung are not known.

Here, we determined the effects of hypercapnia on AT2 progenitor capacity using unbiased RNA-Seq analysis of flow-sorted AT2 cells and validation with lineage-labeled AT2 cells subjected to ex vivo organoid growth. Our findings suggest that hypercapnia limits AT2 cell βcat signaling and progenitor function by altering Wnt expression in surrounding niche cells. We show that hypercapnia skews expression of PDGFRα^+^/stroma-derived Wnt signals away from those typically known to activate βcat signaling (e.g., canonical *Wnt2*) and toward those historically shown to drive morphogenetic processes independently of βcat (e.g., noncanonical *Wnt5a*). We validate this model by showing that WNT5A inhibits βcat signaling in primary AT2 ex vivo cultures. We also show that *Pdgfra/Wnt2* cells are spatially closer to AT2 cells than *Pdgfra/Wnt5a* cells under baseline conditions, where flow-sorted PDGFRα^+^ cells can be subdivided into populations expressing Wnts nonuniformely (e.g., *Wnt2^+^*, *Wnt5a^+^*, *Wnt2^+^/Wnt5^+^*, *Wnt2^–^/Wnt5a^–^*)*,* suggesting that their spatial arrangement could direct distinct AT2 cell behaviors in the distal lung. Importantly, hypercapnia blurs this spatial separation by upregulating *Wnt5a^+^* in *Pdgfra^+^/Wnt2^+^* cells, causing the *Wnt5a^+^* signal to be closer to AT2 cells. These data suggest a mechanism by which hypercapnia may slow lung repair after injury, with broad implications for understanding how stromal cell–derived Wnt signals direct alveolar epithelial behaviors during repair.

## Results

### Hypercapnia limits AT2 proliferation in vitro and in vivo.

**Figure 1:**
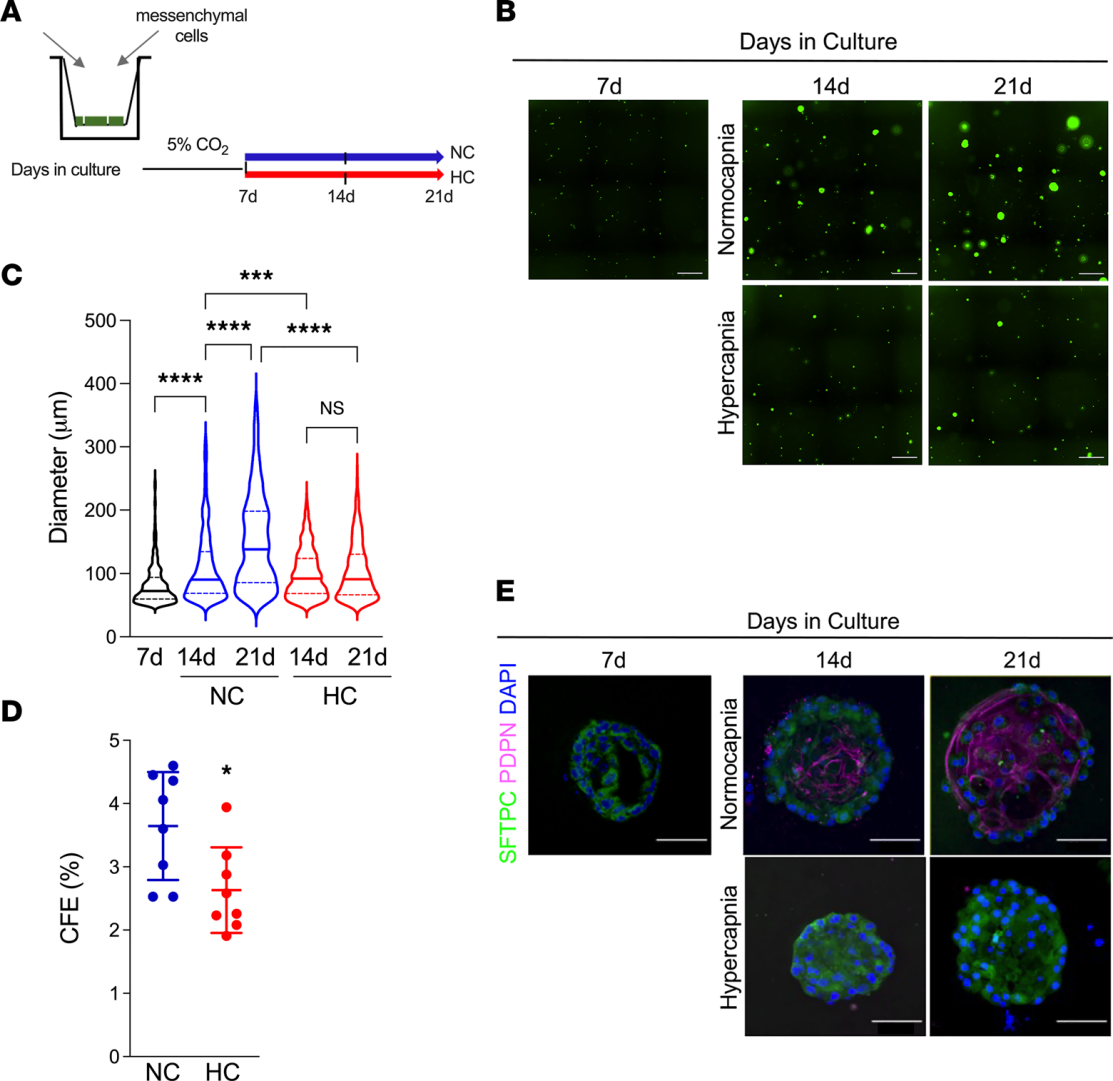
Hypercapnia limits AT2 cell proliferation in 3D culture organoids. (**A**) Schematic of experiments designed to coculture AT2 cells isolated from *Sftp^CreERT2^* *R26R^EYFP^* mice (*Sftpc*^EYFP^ AT2) and WT mesenchymal cells. Alveolar organoids were switched to normocapnia (5% CO_2_; NC) or hypercapnia (20% CO_2_; HC) media on day 7 and cultured until day 21. (**B**) Representative images of organoid cultures in normocapnia or hypercapnia. Scale bars: 500 μm. (**C**) Graph depicts the inhibitory effect of hypercapnia on organoid size. Median with interquartile range. *n* = 8. (**D**) Graph depicts the effect of hypercapnia exposure for 21 days on colony forming efficiency (CFE). *n* = 8. (**E**) Immunofluorescence analysis of SFTPC (AT2 marker) and Podoplanin (AT1 marker) revealed a reduction in AT2 cell proliferation in organoids exposed to hypercapnia for 14 days relative to normocapnia. Nuclear DNA is stained with DAPI. Scale bars: 50 μm. (**C**) ANOVA plus Sidak’s multiple comparisons test. (**D**) Student’s *t* test. *P < 0.05; ***P < 0.001, ****P < 0.0001.

To assess the effect of hypercapnia on AT2 cell progenitor capacity, we employed a 3D organoid model. Mouse primary AT2 cells were isolated by FACS (EPCAM^+^MHCII^+^) from leukocyte/endothelial cell–depleted lung homogenates of WT mice as described (22, 23) (Supplemental Figure 1A; supplemental material available online with this article; https://doi.org/10.1172/jci.insight.159331DS1) or from *Sftp^CreERT2^* *R26R^EYFP^* mice as EPCAM^+^YFP^+^ (Supplemental Figure 1B). In *Sftp^CreERT2^* *R26R^EYFP^* mice*,* tamoxifen administration permanently induces the expression of YFP specifically in AT2 cells and their progeny (18). To establish lung organoids, AT2 cells were embedded in Matrigel in the presence of mesenchymal cells in a 1:10 ratio (Figure 1A). In normocapnic conditions (NC) (5% CO_2_, pH 7.4), sphere-like colony formation was seen typically between 5 and 7 days, which continued for up to 21 days (Figure 1, A and B). When cultures were started in the presence of high CO_2_ (HC) (20% CO_2_, pH 7.4), we did not observe organoid formation (data not shown), suggesting that hypercapnia profoundly affects AT2 cell colony formation. To better assess the consequences of hypercapnia on AT2 progenitor activity, we grew organoids for 7 days in control media before switching cultures to conditioned media equilibrated to either 5 or 20% CO_2_ for an additional 7–14 days (Figure 1, A–E). Under these conditions, small spheres can be observed at 7 days (median diameter, approximately 72 μm) in which AT2 cells have not yet started to differentiate, while larger spheres are present at 14 and 21 days (Figure 1B). Hypercapnia significantly decreased average organoid diameter compared with organoids grown in normocapnia and reduced AT2 clonogenicity (colony forming efficiency [CFE]) (Figure 1, C and D, and Supplemental Figure 2A). Whole organoids were fixed and visualized using Surfactant protein C (SFTPC) and podoplanin (PDPN) to indicate AT2 and AT1 cells, respectively. After 14 and 21 days in culture in NC, organoids displayed a characteristic structure with central AT1 cells surrounded by peripheral AT2 cells; during hypercapnia, few AT2 cells were observed with almost no AT1 cell marker detection (Figure 1E). To determine whether the effects of hypercapnia on AT2 proliferation were reversible, we switched organoids grown under hypercapnia into normocapnia as described in Supplemental Figure 2B. Results show that 7 days of normocapnia can partially reverse the effects of hypercapnia on organoid growth (Supplemental Figure 2, C and D).

**Figure 2:**
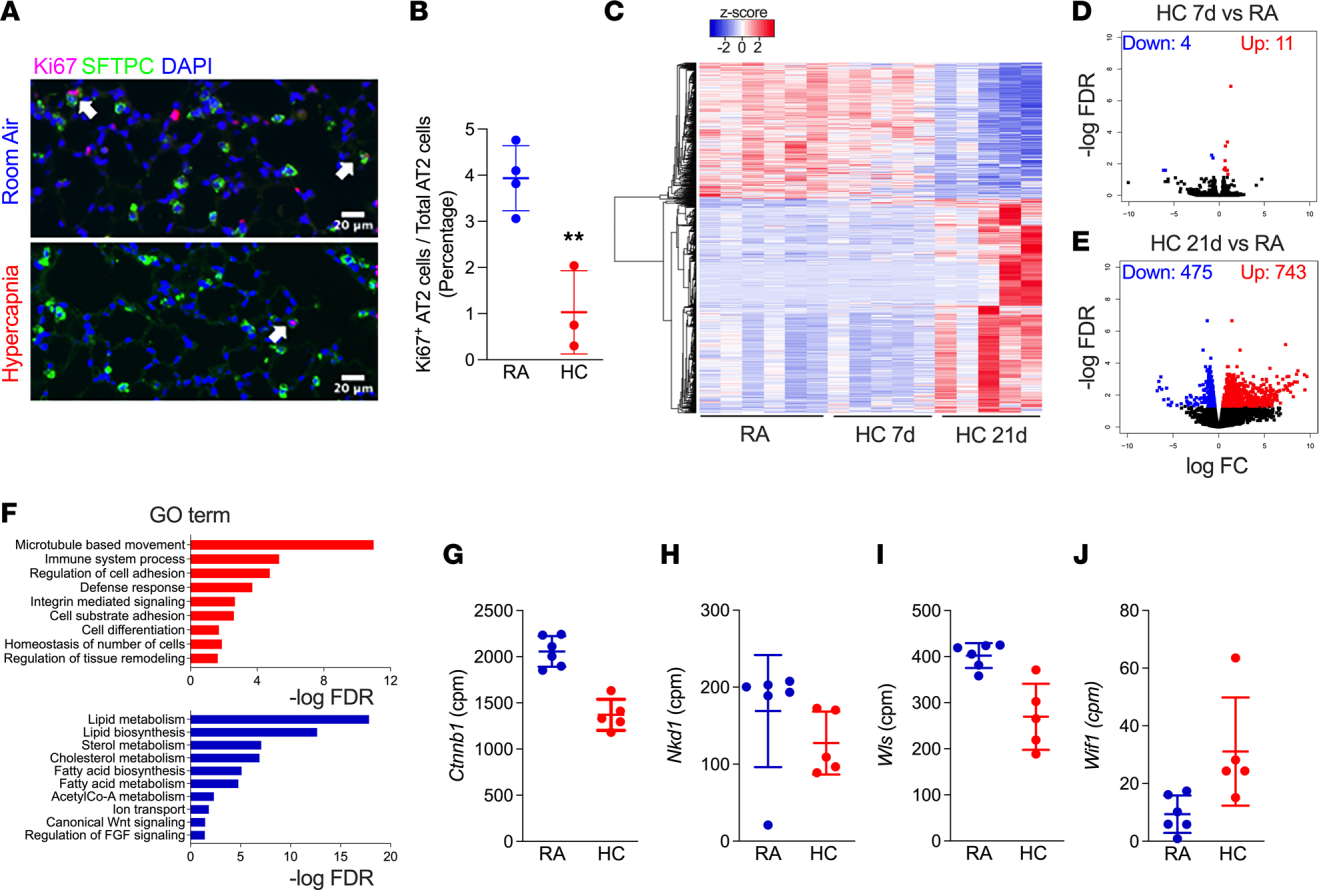
Transcriptomic analysis of isolated AT2 cells reveals inhibition of βcat signaling during hypercapnia. (**A**) Hypercapnia decreases the number of cells expressing Ki67 in the alveolar region of the adult mouse lung exposed to room air (RA) or 10% CO_2_ (HC) for 21 days, as revealed by immunofluorescence. White arrows indicate SPTPC^+^Ki67^+^ AT2 cells. Scale bars: 20 μm. (**B**) Graph depicting the inhibitory effect of hypercapnia exposure for 21 days on proliferation. RA, *n* = 4; HC, *n* = 3 mice. Student’s *t* test. **P < 0.01. (**C**–**F**) Bulk RNA-Seq was performed on flow cytometry sorted AT2 cells from mice breathing RA (*n* = 6) or exposed to HC. Heatmap shows clustering of differentially expressed genes (FDR *q* < 0.05) in AT2 cells after 7 (*n* = 5) or 21 (*n* = 5) days of hypercapnia exposure. (**D** and **E**) Volcano plots. (**F**) GO biological processes. (**G**–**J**) Expression of selected DEG (FDR *q* < 0.05) regulated by hypercapnia involved in the Wnt/βcat pathway.

To assess the effects of hypercapnia on proliferation in vivo, mice were exposed for 21 days to either breathing room air (RA) or 10% CO_2_ (HC). Hypercapnia limited the proliferative capacity of AT2 cells in mice, as evidenced by fewer SFTPC^+^ AT2 cells coexpressing the proliferation marker, Ki67 (Figure 2, A and B).

### Hypercapnia inhibits Wnt/βcat signaling in AT2 cells.

To determine how hypercapnia limits AT2 progenitor capacity and differentiation, we interrogated transcriptional differences in bulk-sorted AT2 cells isolated from mice exposed either to RA or hypercapnia. Mice were exposed to HC for 7 and 21 days and AT2 cells were isolated from single-cell suspensions as described above, RNA was isolated, and bulk RNA-Seq was performed as previously described (24, 25). After 7 days of hypercapnia, only 15 differentially expressed genes (DEG) were identified compared with AT2 cells isolated from mice kept at RA (Figure 2, C and D). After 21 days of exposure to hypercapnia, over 1,200 DEGs were identified compared to room-air mice (Figure 2E). Enrichment analysis of biological processes revealed that hypercapnia inhibits lipid synthesis/metabolism, lysosomal pathways, and canonical Wnt signaling (Figure 2F). Specifically, hypercapnia limited the expression of genes coding for essential regulators of AT2 lineage (*Etv5*, *Abca3*) (26, 27), functional markers of AT2 cell function (*Sftpc*, *Nkx2.1*) (26, 28), and lipid metabolism (*Fabp5*, *Hmgcr*) (26, 28) (Supplemental Figure 3, A–F), suggesting an impairment of AT2 cell maturation and function. Decreased expression of *Spftc/*SFTPC were confirmed at both RNA and protein levels as well as by counting the number SFTPC^+^ cells in lung tissue slides from mice exposed for 21d to hypercapnia or RA (Supplemental Figure 3, G–J). Fibroblast growth factor receptor 2 (*Fgfr2)*, which is necessary for AT2 cell maintenance and self-renewal (29, 30), was decreased by 50% in AT2 cells after 21 days of hypercapnia compared with RA exposed mice (Supplemental Figure 3K), consistent with our evidence that hypercapnia attenuates AT2 proliferative capacity.

**Figure 3:**
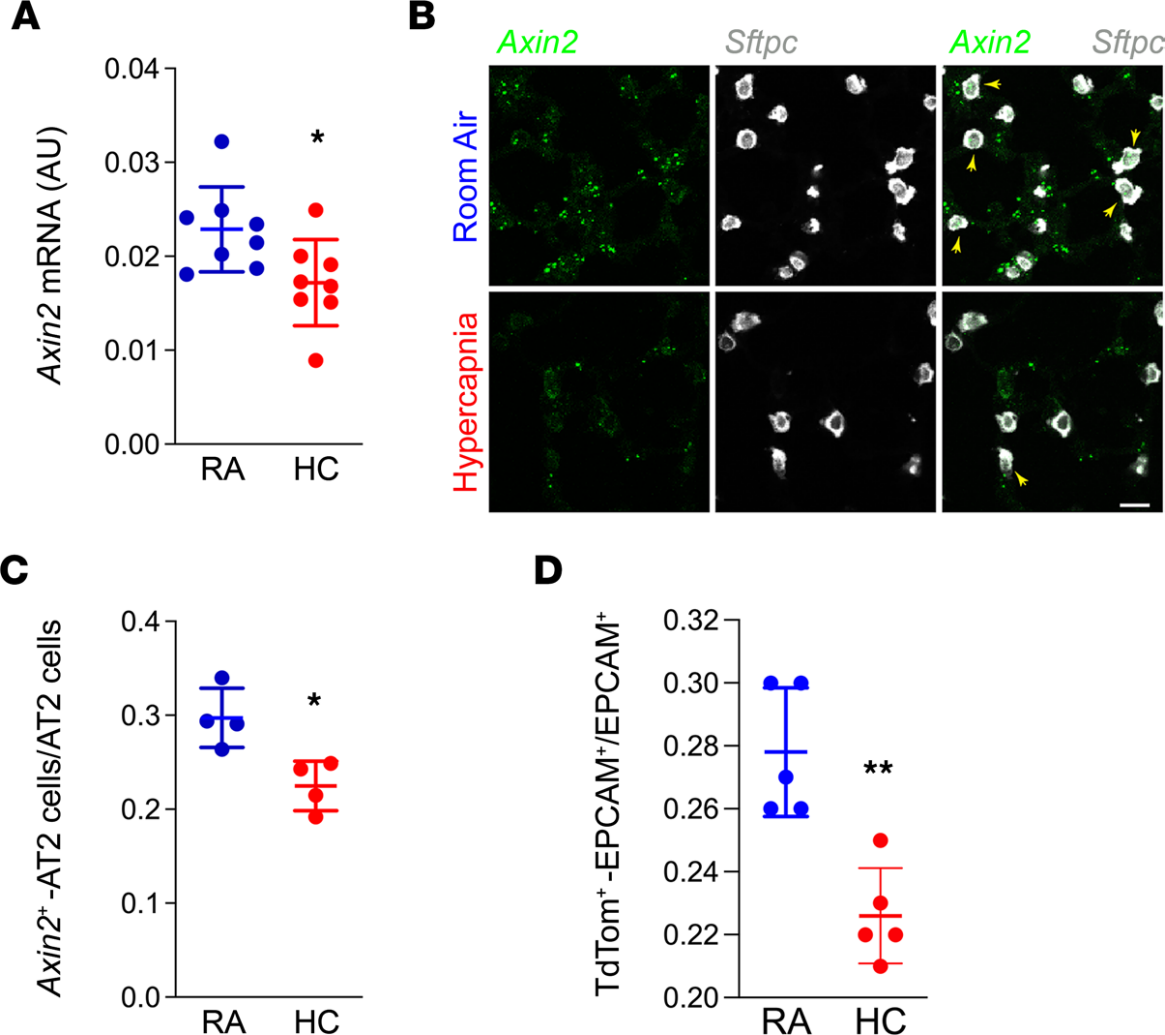
Hypercapnia decreases Wnt/βcat signaling in AT2 cells. AT2 cells were isolated from mice exposed to room air (RA) or 10% CO_2_ (HC) for 21 days. (**A**) mRNA was isolated, and qPCR was performed. *n* = 8 mice. (**B** and **C**) In situ RNA hybridization showing decreased number of *Axin2*^+^ AT2 cells in mice exposed to HC. Yellow arrows indicate *Sftpc^+^Axin2^+^* AT2 cells. Scale bars: 10 μm. *n* = 4 mice. (**D**) Number of lineage-labeled AT2 cells from *Axin2^CreERT2–TdTom^* mice determined by flow cytometry. *n* = 5 mice. Graph shows data from 1 of 3 independent experiments. Student’s *t* test. *P < 0.05; **P < 0.01.

Since Wnt signaling plays a major role in lung homeostasis and repair after injury (18, 19, 31), we interrogated established target genes and components related to this pathway. We found hypercapnia inhibited expression of *Ctnnb1* itself, as well as *Nkd1* (a known target) (32) and *Wls* (required for Wnts secretion) (33), and it increased expression of *Wif1* (34), a negative feed-back regulator of Wnt/βcat signaling (Figure 2, G–J). We confirmed this finding in AT2 cells isolated from mice exposed to hypercapnia for 21 days by monitoring *Axin2* expression, a universal target of Wnt/βcat signaling whose expression reports pathway activation across a range of cell types and tissues (18, 19, 35) (Figure 3A). Hypercapnia also limited the number of *Axin2*-expressing AT2 cells (*Sftpc^+^Axin2^+^*) using multiplexed in situ hybridization of WT mouse lung sections (Figure 3, B and C, and Supplemental Figure 4A) with independent confirmation by using the βcat signaling reporter mouse *Axin2^CreERT2–TdTom^* (Figure 3D and Supplemental Figure 4E). In this Wnt/βcat-responsive fluorescent reporter line, *Axin2*^TdTomato+^ cells are restricted to the distal epithelium and surrounding mesenchyme at E13.5, where this pattern continues through adulthood (18). As with previous reports, we isolated bright populations of *Axin2*^TdTomato–HIGH^–expressing cells that are SFTPC*^–^*, as well as dim *Axin2*^TdTomato–DIM^ SFTPC*^+^* AT2 cells (Supplemental Figure 4, B–D). Together, these data suggest that hypercapnia antagonizes AT2 progenitor activity by limiting βcat signaling within AT2 cells.

### Hypercapnia skews stromal cell–derived Wnts toward a βcat signaling inhibitory environment.

**Figure 4:**
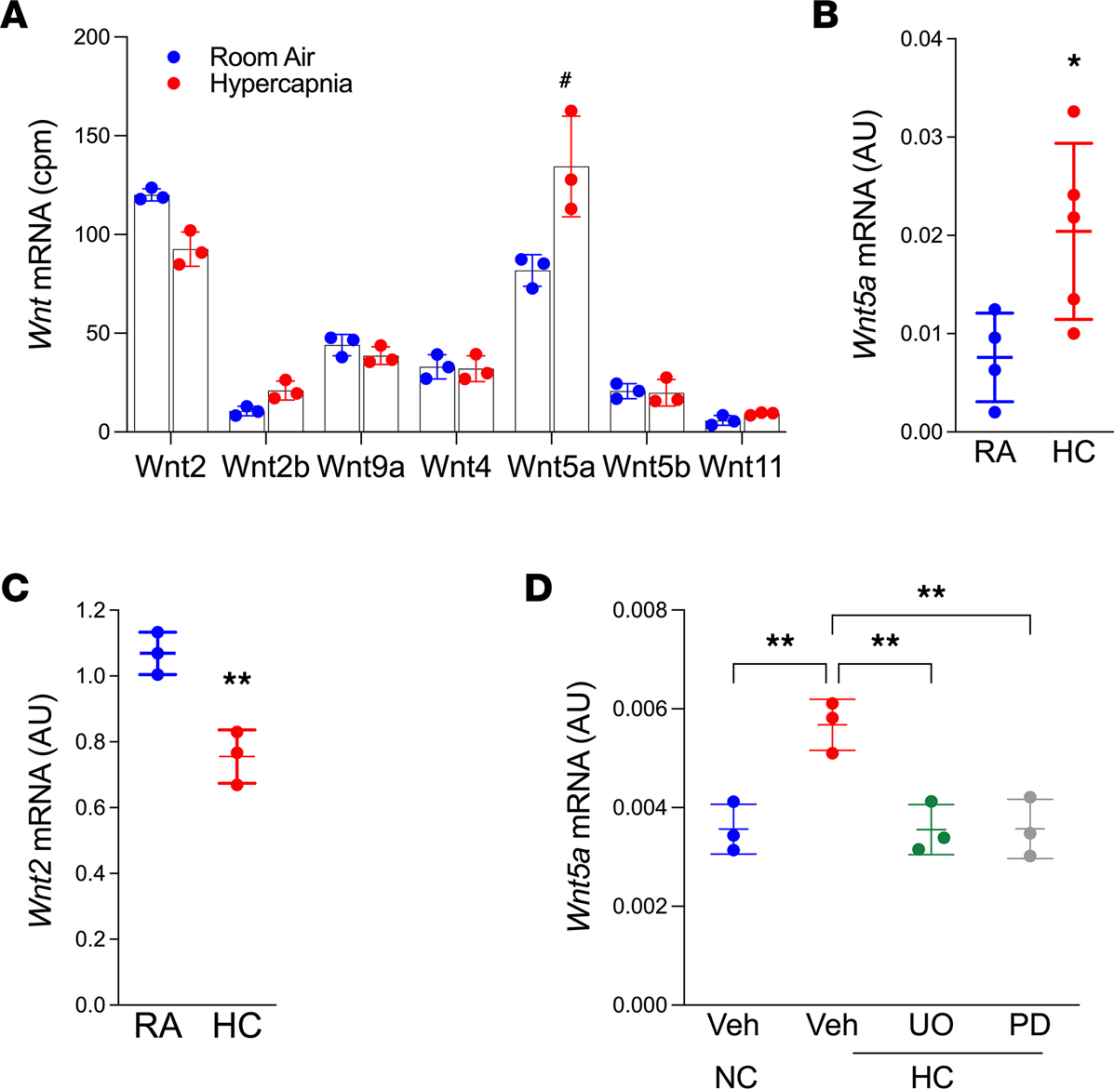
Hypercapnia increases *Wnt5a* expression in PDGFRα^+^ fibroblasts. Lung PDGFRα^+^ fibroblasts were isolated via flow cytometry cell sorting from mice breathing room air (RA) or exposed to 10% CO_2_ (HC) for 10 days. (**A**) Expression of Wnt genes in PDGFRα^+^ fibroblasts as analyzed by population RNA-Seq. *n* = 3, with cells isolated from 3 mice in each replicate. ^#^FDR *q* < 0.05). (**B**–**D**) mRNA was isolated, and qPCR was performed. (**B**) *Wnt5a* (*n* = 4). (**C**) *Wnt2* (*n* = 3). (**D**) MLg2908 mouse lung fibroblast cells were preincubated in the presence or absence of UO126 (10 μM) or PD98059 (10 μM) for 90 minutes and exposed to media equilibrated to NC (5% CO_2_) or HC (20% CO_2_) for 24 hours. *n* = 3. (**B** and **C**) Student’s *t* test. (**D**) ANOVA plus Sidak’s multiple comparisons test. *P < 0.05; ** *P* < 0.01.

To determine the mechanism by which hypercapnia limits βcat signaling activity in AT2 cells, we first interrogated our AT2 bulk RNA-Seq data set for altered expression of Wnt genes, given previous evidence that hyperoxic lung injury upregulates a set of AT2 cell Wnts that could alter “bulk” AT2 behavior (19) and that short-term hypercapnia upregulates Wnt7a in mouse lung homogenates (36). However, *Wnt7b,* one of the most highly expressed AT2 cell Wnts (19), was not altered by hypercapnia (data not shown). While *Wnt3a* and *Wnt4* expression were mildly reduced by hypercapnia, they were minimally expressed and, thus, were not validated by RNA-FISH (not shown) or single-cell data (25). Since stromal niche cells proximal to AT2 cells are critical for AT2 proliferation and differentiation to AT1 cells (13, 16), we sought to assess whether hypercapnia limits alveolar epithelial cell renewal by modifying Wnts produced from adjacent fibroblasts. We isolated PDGFRα-expressing fibroblasts (CD45^–^CD31^–^EPCAM^–^PDGFRα^+^) from mice exposed to HC for 10 days and analyzed their transcriptomic profiles, identifying about 310 genes differentially expressed by exposure to hypercapnia (Supplemental Figure 5). This analysis revealed robust expression of a number of Wnts previously identified in our single-cell RNA-Seq data sets as fibroblast enriched (*Wnt2*, *-2b*, *-9a, -4*, *-5a,* and *-11*) (25), where *Wnt5a* and *Wnt2* were the most abundant (Figure 4A). Hypercapnia significantly increased *Wnt5a*, a Wnt typically known to antagonize βcat signaling and promote cell shape and polarized behaviors (37, 38). Conversely, expression of *Wnt2*, which has been described to activate Wnt/βcat signaling in rat lung fibroblasts in NIH/3T3 (39, 40), trended downwards. The hypercapnia-induced *Wnt5a* increase and *Wnt2* decrease were independently confirmed by isolating mRNA from PDGFRα^+^ fibroblasts (Figure 4, B and C). To interrogate signals downstream of hypercapnia that can enhance *Wnt5a* expression, we preincubated Mlg 2908 mouse lung fibroblasts with pathway inhibitors before exposure to 5% or 20% CO_2_. We found that hypercapnia-induced *Wnt5a* elevation was sensitive to MAPK inhibition, as assessed by the use of PD98059 and UO126 (highly selective inhibitors of MEK1/2 and the MAPK cascade) (Figure 4D). These data are consistent with our previous evidence that acute hypercapnic exposure leads to activation of MAPK-signaling (41), and they suggest that *Wnt5a* may be a target of MAPK signals.

**Figure 5:**
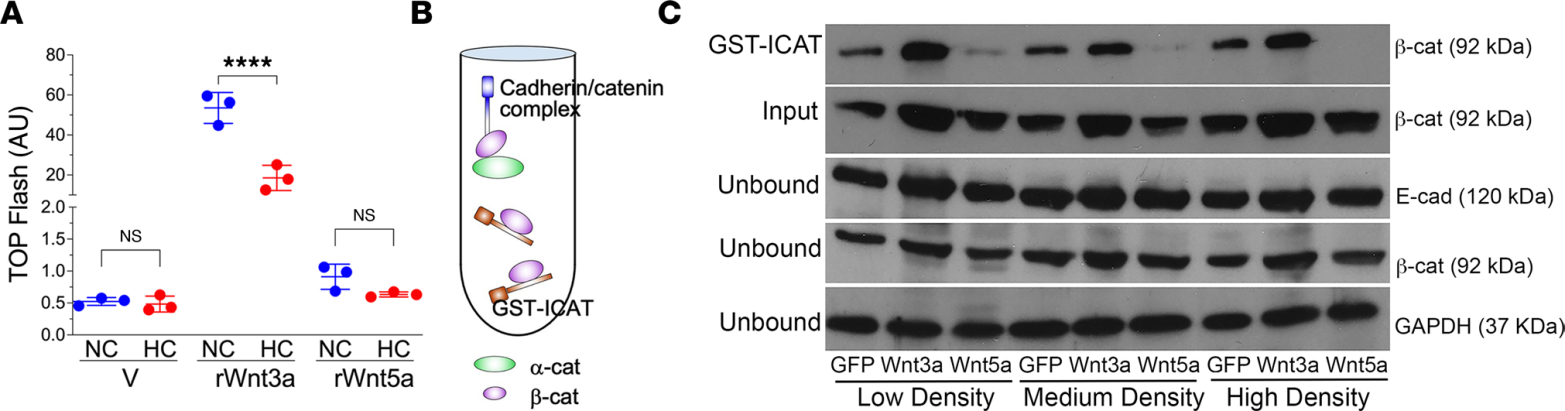
WNT5A reduces βcat signaling in alveolar epithelial cells. (**A**) βCat transcriptional activity was measured using the established, TOPFlash. A549 cells were transiently transfected with the reporter plasmid for 24 hours and were then stimulated with recombinant Wnts for another 16 hours. Cell lysates, normalized to equal protein concentrations, were assayed for luciferase activity. V, vehicle. *n* = 3 independent experiments run in duplicate. (**B**) Schematic for assay to quantify the cadherin-free signaling pool of βcat in cell lysates via GST-ICAT (inhibitor of catenin and T cell factor [TCF]) affinity precipitation as described in Methods. ICAT (brown protein) is an 81 amino acid polypeptide that binds the central armadillo-repeat region of βcat (purple) and can be used to quantify the Wnt-stabilized pool of βcat. Cadherin (blue) and α-catenin (green) are also shown. (**C**) Immunoblot from rat AT2 cells plated at different densities and infected with adenoviruses coding for GFP, WNT3A-IRES-GFP, or WNT5A-IRES-GFP and subjected to GST-ICAT affinity precipitation as in **B**. Input lysates and postaffinity precipitation (unbound lysates) are shown as controls. Note that, across all cell plating conditions, WNT3A increases, whereas WNT5A inhibits the GST-ICAT–bound signal pool of βcat. ****P < 0.0001. One-way ANOVA with Sidak’s post hoc comparison test.

Validating whether particular Wnts are βcat “activating” or “inhibiting” is important, given conflicting reports on the role of WNT5A in AT2 progenitor behavior (19, 37, 42). As is typically observed in established reporter assays, we found that recombinant WNT3A (rWNT3A) promoted βcat signaling in alveolar epithelial (A549) cells, whereas WNT5A did not (Figure 5A). rWNT3A was used as a canonical Wnt/βcat activator (positive control), since validated rWNT2 (i.e., purified from mammalian cell culture secretions) is not available (R&D Systems; Bio-Techne). We independently confirmed these findings by expressing Wnts via adenoviral transduction of primary AT2 cell cultures using an established affinity precipitation–based assay to capture and quantify the cadherin-free signaling fraction of βcat (Figure 5, B and C) (43). This experiment showed that, irrespective of AT2 cell seeding density, WNT5A limits the cytosolic pool of βcat available for signaling (Figure 5C). Conversely, WNT3A elevates the cytosolic pool of βcat in AT2 cells, consistent with its longstanding role as an activator of βcat signaling. Together, these data show that WNT5A antagonizes βcat signaling in cultured AT2 cells and raise the possibility that hypercapnia may limit the progenitor capacity of AT2 cells through stromal cell–derived WNT5A.

### Activation of βcat-signaling rescues AT2 progenitor activity during hypercapnia.

**Figure 6:**
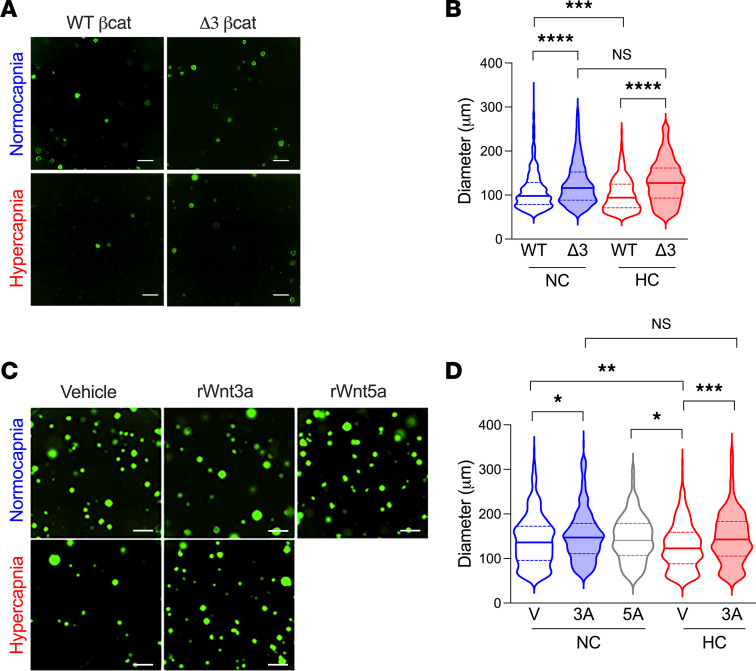
Activation of βcat signaling rescues AT2-proliferative capacity during hypercapnia. (**A**) Representative fluorescence images of typical day 21 organoid cultures of AT2 isolated from *Sftp^CreERT2^* *Ctnnb1^wt/wt^* *R26R^EYFP^* mice (WTβcat) or *Sftp^CreERT2^* *Ctnnb1^flExon3fl^* *R26R^EYFP^* (Δ3βcat). Organoids were cultured in NC or HC as described in Figure 1. Scale bars: 500 μm. (**B**) Graph depicts effect of WTβcat versus Δ3βcat expression on normocapnia (NC) and hypercapnia (HC) organoid size. *n* = 4 mice of each strain in 2 independent experiments. (**C**) Representative fluorescence images of typical day 21 organoid cultures of AT2 cells isolated from *Sftp^CreERT2^* *R26R^EYFP^* mice treated with rWNT3A (50 ng/mL, 3a) or rWNT5A (50 ng/mL, 5a) starting 24 hours after switching media to NC or HC. Scale bars: 500 μm. (**D**) Graph depicts effect of added rWNT5A and rWNT3A on organoid size. *n* = 4 mice of each strain in 3 independent experiments. Data are shown as median with interquartile range. *P < 0.05; **P < 0.01***P < 0.001; ****P <0.0001;. One-way ANOVA with Sidak’s post hoc comparison test.

While hypercapnia can limit βcat signaling activity, the aggregate effects of hypercapnia are likely to be pleotropic. Thus, we sought to test whether the ability of hypercapnia to limit AT2 progenitor capacity could be offset by forced activation of βcat signaling. The most convenient Wnt/βcat signaling pathway activators work by inhibiting glycogen synthase kinase-3β (GSK-3β), the central inhibitory kinase in this pathway (44). GSK-3β inhibitors antagonize βcat phosphorylation and degradation, allowing βcat to accumulate in the nucleus and transactivate Wnt signals. As such, 24 hours after switching the media to hypercapnia, organoids were incubated in the presence or absence of the GSK-3β inhibitor CHIR99021 (CHIR; 20 nM). CHIR did not significantly increase organoid size in normocapnia but significantly rescued organoid growth during exposure to hypercapnia (Supplemental Figure 6, A and B). Since GSK3 has numerous cellular targets with roles in many signaling processes, we also assessed whether the antiprogenitor effects of hypercapnia could be rescued by constitutively activating βcat signaling in AT2 cells using *Sftpc^CreERT2^* *Ctnnb1^Exon3fl/+^* *R26R^EYFP^* mice (45). In these mice, Cre-dependent removal of a phospho-degron in exon3 of the βcat gene (*Ctnnb1*) generates a constitutively active form of βcat. Remarkably, the increase in organoid size by constitutively active βcat was completely refractory to the antiproliferative effects of hypercapnia (Figure 6, A and B). We further asked whether the antiprogenitor effects of hypercapnia could be bypassed by providing exogenous, extracellular Wnt proteins. We found that WNT3A significantly enhanced organoid growth under both normocapnia and hypercapnia conditions, whereas WNT5A limited organoid growth as expected (Figure 6, C and D). Altogether, these data show that the antiprogenitor effects of hypercapnia are reversible and can be offset by elevating βcat signaling within AT2 cells or by adding exogenous Wnts.

### Hypercapnia alters the spatial distribution of Wnt expression in AT2 niche cells.

**Figure 7:**
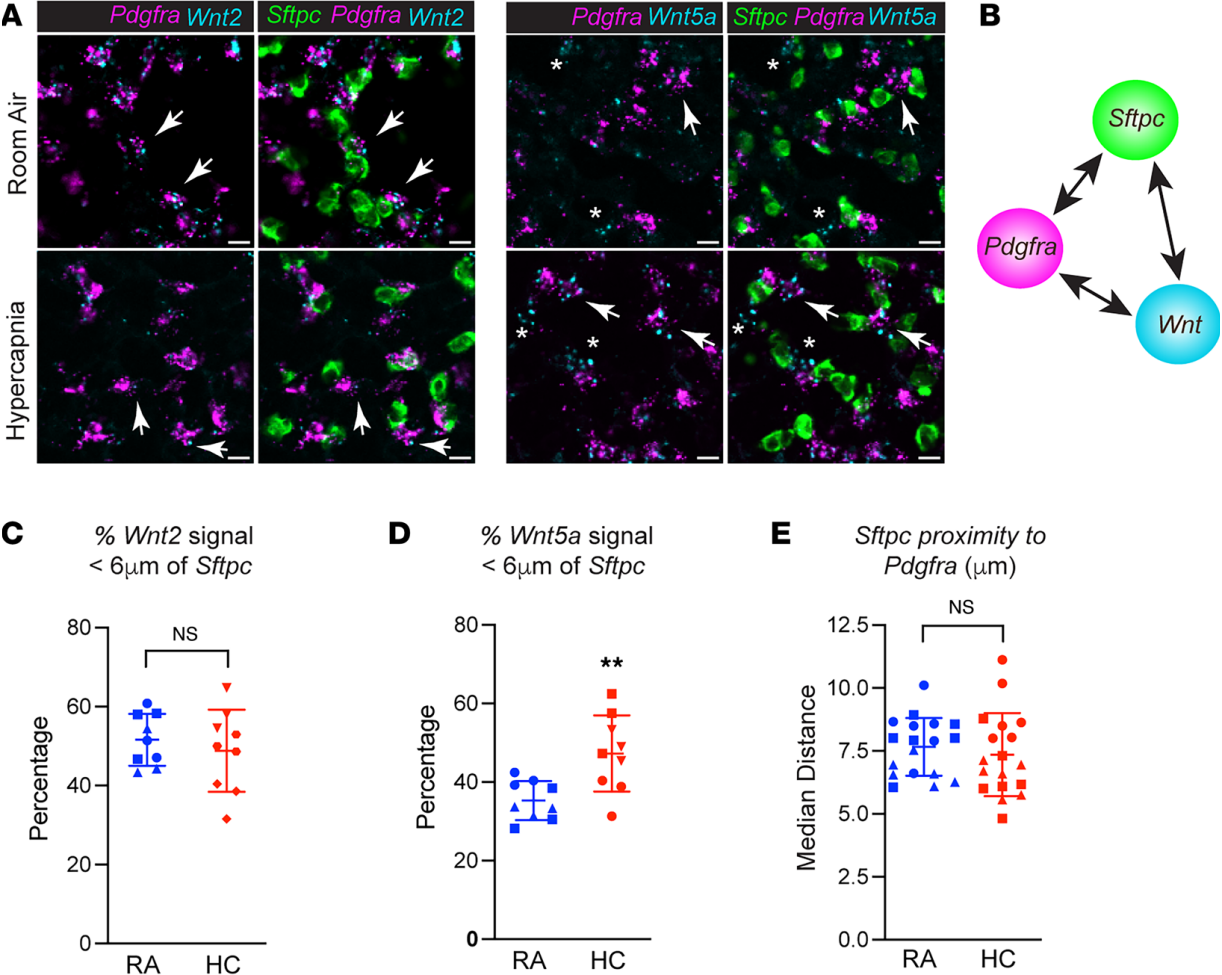
*Pdgfra^+^Wnt2*-expressing fibroblasts are spatially closer to AT2 cells than *Wnt5a*-expressing fibroblasts. (**A**) Confocal images of *Pdgfra, Sftpc, Wnt2*, and *Wnt5a* mRNA signal in lung tissue from WT mice exposed to room air (RA) or hypercapnia (HC) for 10 days. RNA-FISH signal intensity converted to object spots to measure shortest distance between signals. White arrows indicate cells coexpressing signals. Scale bars: 10 μm. (**B**) Schematic of spatial distance mapping algorithm. (**C** and **D**) Graphical representation of the percentage of *Wnt2* or *Wnt5a* spots from *Sftpc* mean signal, respectively. *n* = 3 (each point consists of 3 mice), 3 fields of view/mouse with more than 1,500 measurements per condition. Student’s *t* test. **P < 0.01. (**E**) Graph shows that hypercapnia does not alter the median distance between *Pdfgra* and *Sftpc* signals.

Given recent evidence that the AT2 cell niche may comprise as few as 1–2 stromal cells to modulate βcat signaling in AT2 progenitor cells (19, 21) and to address how hypercapnia limits AT2 progenitor function, we sought to measure the spatial proximity of *Wnt2* and *Wnt5a* signal to *Sftpc*^+^ AT2 cells RNA-FISH. We focused on *Wnt2* for comparison with *Wnt5a*, since it is one of the most abundant stromal cell–derived Wnts (21, 25) and is known to activate βcat signaling across diverse cell types (46, 47). Given that WNT5A can antagonize the βcat signaling pool in AT2 cells (Figure 5), we reasoned that βcat-activating and -inhibiting Wnts might be spatially separated in the AT2 niche to control AT2 progenitor versus differentiation decisions. By converting RNA-FISH signal to objects (i.e., “spots”) based on signal intensity, we measured median shortest distances between signal pairs (Figure 7, A and B). We found that, while both *Wnt2*- and *Wnt5a*-expressing *Pdgfra^+^* stromal cells are spatially proximal to *Sftpc^+^* AT2 cells, in normocapnic cells, the *Wnt2* signal is significantly closer than *Wnt5a* (40 vs. 30% < 6 μm, respectively; Supplemental Figure 7). We next asked whether the spatial proximity of these Wnts was altered by hypercapnia. Remarkably, we found that hypercapnia led to a greater percentage of *Wnt5a* signal within the first distance bins (6 μm), while the *Wnt2* signal did not significantly change (Figure 7, C and D). As expected, hypercapnia did not change the proximity of *Pdgfra* signal to *Sftpc* (Figure 7E). Collectively, these data suggest that hypercapnia increases *Wnt5a* expression, particular within the AT2 cell proximal zone normally occupied by *Wnt2*.

**Figure 8:**
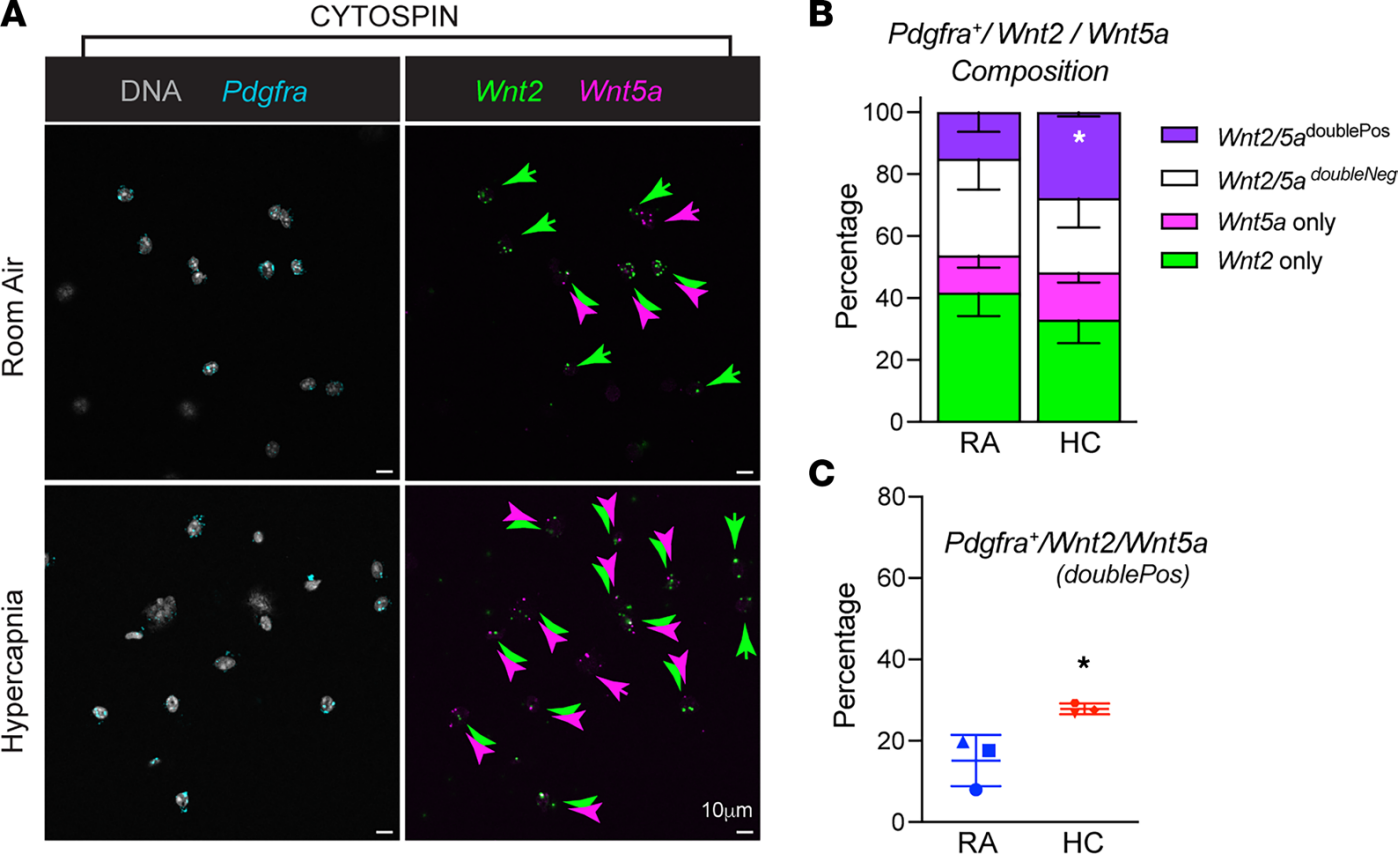
PDGFRα^+^ flow-sorted fibroblasts show nonuniform expression of *Wnt2* and *Wnt5a*. (**A**) Confocal images of PDGFRα flow-sorted fibroblasts isolated from room air (RA) and hypercapnia (HC) exposed mice for 10 days were subjected to cytospin/RNA-FISH analysis with probes for *Pdgfra* (cyan), *Wnt2* (green), and *Wnt5a* (magenta). Nuclei are shown in gray. RNA-FISH signal intensity converted to object spots to measure cooccurrence of *Wnt2, Wnt5a*, or both signals within the *Pdgfra* signal region. Colored arrows denote single-positive Wnt cells and double-positive Wnt cells. Scale bars: 10 μm. (**B** and **C**) Graphical representation of the percentage of *Wnt2* or *Wnt5a* single-positive versus double-positive *Pdgfra^+^* cells. *n* = 3 (each point consists of 3 mice), 3 fields of view/mouse with more than 400 measurements per condition. *P < 0.05.

To further interrogate our model of spatially separated βcat-activating versus βcat-inhibitory Wnts, we asked whether *Pdgfra*^+^ stromal cells are uniformly *Wnt2^/^Wnt5a*-double positive or instead comprise Wnt heterogeneity. For this, we used an imaging method that would allow visualization of the entire *Pdgfra^+^* cell population, rather than being limited to a 15 μm thickness of frozen lung sections. We isolated PDGFRα^+^ cells by flow cytometry and performed RNA-FISH analysis after cytospin. Interestingly, we found that the PDGFRα^+^ cells could be distinguished as 4 subtypes regarding *Wnt2/Wnt5a* expression: *Wnt2^+^*, *Wnt5a^+^*, *Wnt2/Wnt5a* double positive, and *Wnt2/Wnt5a* negative (Supplemental Figure 8 and Figure 8A). While PDGFRα^+^ cells no doubt express other Wnts, the results show that this population is not uniformly positive for both *Wnt2* and *Wnt5a*. Together with the evidence that these Wnts show different average distances from AT2 cells in lung sections, these data suggest that PDGFRα^+^ stromal cells establish a spatial code of βcat-activating versus βcat-inhibitory Wnt ligands to narrowly control the AT2 cell proliferative zone of the alveolus. We then sought to determine whether hypercapnia could alter the relative abundance of *Wnt2/Wnt5a/Pdgfra* subsets by performing RNA-FISH analysis on flow-sorted PDGFRα^+^ cells harvested from 3 independent mice subjected to hypercapnia versus RA. We observed a significant increase in the *Wnt2^+^/Wnt5a^+^* subpopulation, which may come at the expense of *Wnt2^+^* and *Wnt2^–^Wnt5a^–^* subpopulations (Figure 8, B and C). Thus, AT2 cells reside within a niche PDGFRα^+^ stromal cells that are heterogenous regarding *Wnt2* and *Wnt5a* expression. Since *Pdgfra^+^/Wnt2*^+^ and *Pdgfra^+^/Wnt2^+^/Wnt5a*^+^ appear spatially separated (albeit over a narrow distance range), where hypercapnic injury reduces the spatial separation of *Wnt2* versus Wnt5a RNA signals proximal to AT2 cells, we reason that hypercapnia disrupts the spatial Wnt code of the AT2 niche.

## Discussion

**Figure 9:**
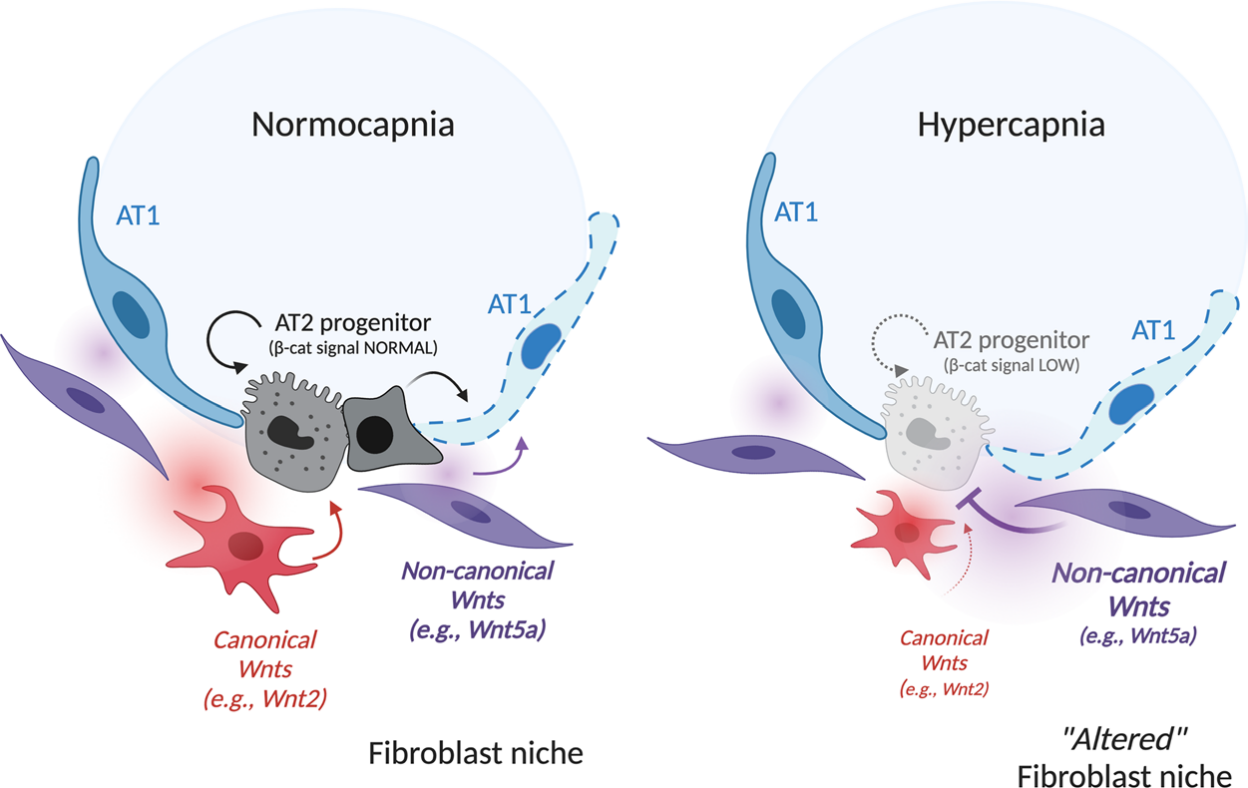
Model for hypercapnia-mediated inhibition of βcat signaling in AT2 cells via skewing of fibroblast-derived Wnts. At the left, normocapnia is represented. AT2 progenitors are spatially proximal to *Pdgfra/Wnt2*-expressing fibroblasts (red/red gradient) that maintain βcat signaling and AT2 self-renewal to replace damaged AT1 cells. *Pdgfra/Wnt5a-*expressing fibroblasts (purple/purple gradient) are spatially farther from the AT2 cell, perhaps to ensure separation of competing βcat-activating (WNT2) from βcat-inhibiting (WNT5A) signals. At the right, hypercapnia leads to reduced βcat signaling in AT2 cells, impairing cell renewal and differentiation by skewing Wnt expression in PDGFRα stromal cells toward a noncanonical variety, with *Wnt5a* significantly elevated. Narrowness of the AT2 progenitor niche raises the possibility that elevated WNT5A release (purple gradient) in close spatial vicinity to the WNT2 signal (red gradient) antagonizes βcat signaling in AT2 cells, inhibiting proliferative capacity.

We describe a mechanism by which hypercapnia, an inevitable consequence of a lung protective ventilation strategy, slows or prevents lung repair after injury. We found hypercapnia decreases AT2 cell progenitor activity by modulating the repertoire of Wnt signals in mesenchymal cells comprising the AT2 stem cell niche. Specifically, hypercapnia reduced expression of Wnt ligands that promote βcat signaling in AT2 cells, and it simultaneously enhanced expression of Wnt ligands that inhibit βcat signaling. These changes affected the AT2 cell microenvironment, leading to reduced AT2 proliferation. Our results suggest that pathophysiological conditions that impact the AT2 niche, including hypercapnia, can disrupt signals from the mesenchyme required to restore the alveolar barrier function and lung homeostasis after injury (Figure 9).

Previous lineage tracing experiments show that a subgroup of AT2 cells, while retaining their surfactant biosynthetic capacity, can act as facultative progenitors (19). Our experiments suggest that the proliferative capacity of AT2 cells is decreased by hypercapnia. In a 3D organoid model, the inhibitory effects of hypercapnia on the proliferative capacity of AT2 cells are so severe that, if the organoid incubation is started in hypercapnia equilibrated media, no organoids are formed. The decrease in AT2 proliferative capacity was also observed in lungs isolated from mice exposed to HC for 21 days. To identify signaling pathways responsible for the hypercapnia-mediated decrease in proliferative activity, we analyzed the transcriptomic signature elicited in AT2 cells isolated from mice breathing RA or HC. We observed changes in the transcriptomic signature at 21 but not at 7 days of hypercapnia. At 21 days, hypercapnia decreased canonical AT2 markers, including *Sftpc* and genes involved in lipid metabolism (*Hmgcr*, *Fabp5*), as well as essential regulators of AT2-cell specification, such as *Etv5* and *Abca*. We also observed an increase in the expression of genes involved in cell adhesion, tissue remodeling, and cytoskeleton reorganization. Together, these data suggest hypercapnia causes a loss of AT2 cell morphological as well as functional characteristics.

Accumulating evidence suggests that canonical Wnt signaling is essential to allow alveolar progenitor cells to undergo alveolar epithelial repair after injury (18, 19, 31). The ubiquitous Wnt/βcat target gene and negative feedback regulator *Axin2* marks a subpopulation of Wnt-responsive AT2 cells with progenitor characteristics (16, 19). Consistent with previous observations, we found that Wnt/βcat activity (evidenced by *Axin2* expression) is very low in control AT2 cells. A recent publication suggests that *Wnt5a*/WNT5A expressed by fibroblasts in close proximity to AT2 cells can induce canonical Wnt signaling, promote AT2 cell cycle entry, and maintain the AT2 cell fate (19). While these activities are typically attributed to elevated βcat signaling, and WNT5A can promote βcat signaling in certain contexts (48), our data reinforce a model in which WNT5A limits βcat signaling in AT2 cells. We validated WNT-subtype functionality in primary AT2 cultures, showing that WNT5A limits, whereas WNT3A enhances, βcat protein levels and signaling. Our results align with a recent lung organoid study showing that WNT5A and WNT5B ligands inhibit alveolar epithelial stem/progenitor expansion by impairing canonical Wnt signaling (38) as well as longstanding evidence that WNT5A limits βcat signals in lung and other tissues (49, 50). Our data are also consistent with evidence that the mesenchymal population that sustains the self-renewal and differentiation of AT2 stem cells is positive for *Wnt2* and other genes (21).

Our results highlight the importance of methods that provide single-cell spatial resolution in studies of lung repair. Spatial transcriptomic approaches allowed us to localize these signals specifically to mesenchymal cells near AT2 cells. We found that freshly isolated PDGFRα^+^ cells can be distinguished as 4 subtypes. These data also suggest AT2 niche cells (i.e., PDGFRα stromal cells) may be targets of pathological conditions like hypercapnia, where the spatial arrangement of distinct PDFGRα^+^ stromal cell subtypes may direct AT2 cell proliferative versus differentiative zones of the alveolus. Using RNA-FISH analysis, we found that *Wnt2*-expressing *Pdgfr*α*^+^* cells are spatially closer to *Sftpc^+^*AT2 cells than *Wnt5*a-expressing cells. These data suggest that a more systematic evaluation of the major stromal cell–derived Wnts may shed light on how individual AT2 cells are selected for activation.

Our results do not exclude pathogenic effects of hypercapnia on AT2 cells independently of the niche. Indeed, hypercapnia can inhibit proliferation of both lung epithelial (A549 human lung carcinoma) and fibroblast (N12) cell cultures (8). As the former is known to express WNT5A (51, 52), future studies will be required to distinguish whether the anti-proliferative effects of hypercapnia are due to WNT5A or other targets in these cells.

In summary, we demonstrate a fundamental effect of elevated CO_2_ levels on alveolar epithelial cell behavior, which is regulated by Wnt signal modulation that inhibits reparative AT2 progenitor cell function. These findings are of biological and clinical relevance, as they pertain to patients with severe COVID-19 ARDS requiring mechanical ventilation. Inhibition of AT2 proliferation in patients with hypercapnia prevents the sealing of the epithelial barrier, increasing lung flooding, ventilator dependency, and mortality. We contend that the hypercapnia-mediated mechanisms we uncovered are of relevance to lung repair, worsening the outcome of mechanically ventilated patients.

## Methods

### Mouse strains and Cre recombinase induction.

All strains including WT mice were bred and housed at a barrier- and pathogen-free facility at the Center for Comparative Medicine at Northwestern University. Animal experiments were performed on both male and female animals in all conditions, and animals were chosen at random from the cohort but not formally randomized. Adult (8–10 weeks old) C57BL/6J mice were obtained from The Jackson Laboratory (strain no. 000664) and were used as the WT strain. *Sftpc^CreERT2^* *R26R^EYFP^* and *Axin2^CreERT2–TdTom^*,Ctnnbl^fl(ex3)/+^ were provided by Edward E. Morrisey (University of Pennsylvania), and their genotyping and characterization has been previously described (18, 45, 53).

For induction of estrogen-inducible Cre recombinase (Cre-ERT2) for conditional tissue–specific conditional alleles in vivo, tamoxifen was dissolved in sterile corn oil (MilliporeSigma, T5648) at 20 mg/mL concentration. Mice were injected i.p. 3 times over the course of 5 days with 0.25 mg/g body weight to induce Cre recombination of floxed alleles for lineage tracing in *Sftpc^CreERT2^* *R26R^EYFP^* (18).

Mice were provided with food and water ad libitum, maintained on a 14-hour light/10-hour dark cycle. For HC exposure, mice were maintained at 10% CO_2_ in a BioSpherix C-Shuttle Glove Box (BioSpherix) for up to 21 days as described previously (54). Control mice were maintained in the adjacent space under RA conditions. We have previously shown that mice exposed to HC had elevated PaCO_2_ and higher bicarbonate values after 3 days of exposure, reflecting renal compensation of the respiratory acidosis (55, 56). Treatment of mice with 10% CO_2_ produces an arterial partial pressure of CO_2_ (pCO_2_) of about 77 mmHg, which is not unusual in patients undergoing “permissive hypercapnia” mechanical ventilation, patients with COPD, or patients with a severe asthma attack (54). At the selected time points, mice were euthanized with Euthasol (pentobarbital sodium and phenytoin sodium) and the lungs were harvested.

### Mouse AT2 cell isolation by flow cytometry.

Tissue preparation for mouse AT2 isolation was performed as described (22, 23), with modifications. Multicolor flow cytometry and cell sorting were performed with an LSR Fortessa or BD FACSAria cell sorter using BD FACSDIVA software (BD Biosciences). Briefly, perfused lungs were treated with 50 U/mL dispase (Corning, 47743-724) and 0.25 mg/mL DNase (Sigma-Aldrich, D4513-1VL) and were subjected to manual dissection gently tearing and mincing the lung pieces. When required, single-cell suspensions were enriched for epithelial cells using anti-EpCAM magnetic microbeads (Miltenyi Biotec, 130-105-958; lot 5210607845).

Lungs from WT, *Sftpc^CreERT2^* *R26R^EYFP^,* and *Axin2*^CreERT2–TdTomato^ mice were processed into single-cell suspensions as indicated above. WT AT2 cells: AT2 cells were sorted from the single-cell suspensions using antibody staining for CD31-PECy7, clone 390 (eBioscience, 25-0311-81; lot 2313111), CD45-PECy7 clone 30-F-11 (eBioscience, 25-0451-82; lot 238036), and EpCAM-APC (eBioscience, 17-5791-80; lot 2202308). WT AT2 cells CD45^−^CD31^−^EpCAM^+^ cells were gated for MHCII-BUV395 clone 2G9 (BD Bioscience, 743876; lot 02970248) and selected as EPCAM^int^MHCII^hi^ as previously described (57) and shown in Supplemental Figure 1. In *Sftpc*^EYFP^ AT2 cells, following negative selection for CD31 and CD45, YFP^+^ AT2 cells were isolated based on enhanced YFP (EYFP) fluorescence and sorted as indicated above and in Supplemental Figure 1. In *Axin2*^TdTom^ AT2 cells, following negative selection for CD31 and CD45, *Axin2^+^* AT2 cells were positively gated for TdTomato and EpCAM as indicated above and in Supplemental Figure 4. Compensation, analysis, and visualization of the flow cytometric data were performed using FlowJo software (FlowJo, 10.7.1). Fluorescence minus one controls were used to set up gates.

### Alveolar organoids and hypercapnia exposure.

Clonal alveolar organoid assays were performed as described previously (13, 18). In brief, a mixture of AT2 cells (5 ***×*** 10^3^ AT2, YFP^+^ AT2, or YFP^+^ βcat ΔExon 3) and lung fibroblasts (5 ***×*** 10^4^) in growth media containing α-MEM media (Thermo Fisher Scientific, 41061029) supplemented with 4.5 g/L D-glucose, 2 mM L-glutamine, 10% FBS, 1% penicillin-streptomycin, 1% insulin/transferrin/selenium (Thermo Fisher Scientific, 41400045), 0.002% Heparin, 0.25 μg/mL Amphotericin B (MilliporeSigma, A2942), and 2.5 μg/mL ROCK inhibitor Y24632 (Selleckchem, S1049) was used for the assays. Lung fibroblasts for organoids assays were isolated from adult WT mice and plated in DMEM supplemented with 4.5 g/L D-glucose, 2 mM L-glutamine, 10% FBS, and 1% penicillin-streptomycin as previously described (16). Immediately before use, cells were treated with mitomycin-C (MilliporeSigma, M4287) for 2 hours. Cells were then suspended in a 1:1 mixture of organoid growth media and growth factor–reduced phenol-free Matrigel (Corning, 356231). In total, 100 μL of the cell/media/matrigel mixture was then plated into individual 24-well cell culture inserts (Corning, 3470) and allowed to solidify at 37°C for 5 minutes. Organoid growth media were added into the bottom of the 24-well plate. After 5–7 days of culture, organoids were moved to normocapnic or hypercapnic conditions and grown for an additional 7–21 days.

We have previously described that the maximal effects of hypercapnia on signal transduction pathways is achieved at ~120 mmHg of CO_2_ (8, 58); all the in vitro experiments in this publication were performed under those conditions. The desired CO_2_ and pH levels were achieved by equilibrating the medium overnight in a humidified chamber (C-Chamber, BioSpherix). The atmosphere of the C-Chamber was controlled with a PRO CO_2_ controller (BioSpherix). In this chamber, cells were exposed to the desired pCO_2_ while maintaining 21% O_2_ balanced with N_2_. Routinely, before CO_2_ exposure, pH, pCO_2_, and pO_2_ levels in the medium were measured using a Nova Stat Profile Prime CCS (Critical Care System; Nova Biomedical). Experiments began by replacing the culture medium with the CO_2_-equilibrated medium and incubating in the C-Chamber for the desired time (59). The normocapnia growth media consisted of α-MEM/Ham’s F-12 medium/Tris base/MOPS base (3:1:0.25:0.25) containing 10% FBS, 100 U/mL penicillin, and 100 μg/mL streptomycin solution (pH 7.4) as we previously described (54, 60). The buffering capacity of the medium was achieved by changing its initial pH with Tris and MOPS base to obtain a pH of 7.4 at the experimental CO_2_ levels (pCO_2_ of 40 or 120 mmHg).

The organoid media with or without treatment was changed every 48 hours. Organoids were imaged on a Nikon Ti^2^ Widefield in bright-field and GFP channels. In some cases, at the end of the experiment, organoids were fixed in 4% paraformaldehyde. Images were processed in Nikon Elements (5.11.00) and quantified using ImageJ/Fiji software (NIH) for organoid diameter. Alveolar organoids are defined as a clonal colony with a minimum diameter of 50 μm. The following antibodies were used: PDPN (DSHB University of Iowa, 8.1.1; 1:40), SFTPC (MilliporeSigma; AB3786, lot 3845252; rabbit polyclonal antibody; 1:250), and imaging was performed in a Nikon Eclipse Ti confocal microscope.

### Immunostaining of whole lung sections.

After lungs were cleared of blood by perfusion with cold PBS via the right ventricle, they were inflated with 4% paraformaldehyde and allowed to fix overnight. Tissue was then dehydrated, paraffin embedded, and sectioned. H&E staining was performed to examine morphology. IHC was used to detect protein expression using the following antibodies on paraffin sections: SFTPC and Ki67 clone SolA15 (eBioscience, 14-5698-8; rat monoclonal antibody; 1:100). Following PBS washes, fluorophore-conjugated secondary antibodies were prepared at 1:500 for 60 minutes in the dark at room temperature. Hoechst 33342 (Invitrogen, 3570) was diluted 1:1,000 and applied for 15 minutes at room temperature following secondary antibody incubation. Coverslips and filters were mounted using ProLong Gold antifade solution. Images were obtained using Zeiss Axioplan epifluorescence. Cell counts were performed using ImageJ software (NIH).

### Isolation of mouse lung fibroblasts.

Mesenchymal populations from whole lung were isolated from WT C57BL/6 mice. Briefly, perfused lungs were inflated with digestion buffer containing 200 U/mL Collagenase (MilliporeSigma, C0130-500 mg); 4 U/mL Elastase (Worthington, LS002292), 0.25 mg/mL DNase (MilliporeSigma, D4513-1VL) and coarsely minced before processing in C-tubes (Miltenyi Biotec) with a GentleMACS dissociator (Miltenyi Biotec) according to the manufacturer’s instructions. Homogenate was passed through 40 μm nylon filter to obtain a single-cell suspension and subjected to RBC lysis reagent (BD Pharm Lyse, BD Biosciences). Cells were subjected to negative selection using CD45 and CD31 magnetic microbeads (Miltenyi Biotec, 130-052-301 [lot 5220509667] and 130-097-418 [lot 521101846]) and live cells selected using Sytox Green (Invitrogen, 2420608). Fibroblasts cells were identified as CD45^–^CD31^–^EPCAM^–^PDGFRA^+^ by flow cytometry as outlined in Supplemental Figure 5 using PDGFRA antibody (eBioscience, 12-1401-81; lot 2124709).

### Transcriptomic profiling via RNA-Seq.

FACS-based isolation of AT2 cells or PDGFRα^+^ fibroblasts from WT mice was performed at the indicated time points for each experiment as described above. The RNeasy Plus Micro Kit (Qiagen, 74034) was used to isolate RNA and remove genomic DNA. RNA quality was assessed with the 4200 TapeStation System (Agilent Technologies). Samples with an RNA integrity number (RIN) lower than 7 were discarded. RNA-Seq libraries were prepared from 5 ng total RNA using the NEB Next RNA Ultra Kit (QIAGEN) with poly(A) enrichment. Libraries were quantified and assessed using the Qubit Fluorimeter (Invitrogen, Thermo Fisher Scientific) and the Agilent TapeStation 4200. Libraries were sequenced on the NextSeq 500 instrument (Illumina) at 75 bp single-end reads. The average reading depth across all experiments exceeded 6 × 10^6^ per sample, and more than 94% of the reads had a Q score above 30. Bioinformatics work was performed on “Genomics Nodes” using Northwestern High-Performance Computing Cluster, Quest (Northwestern IT and Research computing). Reads were demultiplexed using bcl2fastq (version 2.17.1.14). Quality was controlled with FastQC. Samples that did not pass half of the 12 assessed quality control (QC) statistics were eliminated. Then, reads were trimmed and aligned to mm10 reference genome using TopHat2 aligner (version 2.1.0). Read counts were associated with genes using the GenomicRanges (61). The downstream differential gene expression was conducted with edgeR/Bioconductor packages (62, 63). Genes with less than 1 normalized counts from more than 3 samples were excluded from the analysis. Hierarchical clustering heatmaps were also performed for the top 1,000 most differential expressed genes. Statistical significance was controlled at a *P* value of 0.05 with FDR adjusting for multipairwise comparison. GO analysis was performed using GOrilla (64) on 2 unranked gene lists.

### qPCR.

RNA was isolated from primary AT2 cells or PDGFRα^+^ fibroblasts or cultured fibroblast cell line (Mlg 2908-CCL-206, ATCC) and purified using RNeasy Plus Micro Kit before cDNA preparation with qScript cDNA synthesis kit (Quanta Bio, 95047). For quantitative PCR (qPCR), IQ SybrGreen master mixes (Bio-Rad, 1708880) were used according to manufacturer’s instructions. The following primers used are described in Supplemental Table 1. TaqMan qPCR was performed for Wnt2 using predesigned TaqMan primer/probe sets (Invitrogen, assay ID: Mm01156901) using Gapdh (assay ID: Mm99999915_g1) following manufacturer’s instruction. qPCR was performed in triplicate for each biological sample, and fold enrichment was calculated from ΔCt values for each gene of interest, normalized to expression of housekeeping genes.

### Immunoblotting.

Protein content in cell lysates was quantified by Bradford assay. Equal amounts of proteins were resolved on polyacrylamide gels (SDS-PAGE). Proteins were transferred to a nitrocellulose membrane using a Trans-Blot-Turbo Transfer System (Bio-Rad). Incubation with specific primary antibodies was performed overnight at 4°C. SFTPC was detected using MilliporeSigma (rabbit polyclonal antibody AB37086; 1:2,500) and actin using a rabbit polyclonal antibody from Sigma-Aldrich (catalog 2066; lot 018M4753V; 1:2,000). HRP-tagged secondary antibodies (Bio-Rad, 1721011 and 1721019) were used in combination with Super-Signal ECL kit (Thermo Fisher Scientific) to develop blots using a LI-COR Fc Odyssey Imager and companion software Image Studio version 5.2. Blots were quantified by densitometry (ImageJ 1.46r).

### Single-molecule RNA-FISH.

Multiplex fluorescence in situ hybridization was performed using RNAscope (Advanced Cell Diagnostics [ACD]). Mouse lungs were inflated to 15 cm H_2_O and fixed with 4% paraformaldehyde (EMS) for 24 hours. Lungs were paraffin embedded, and 5 μm tissue sections were mounted on Superfrost Plus slides (Thermo Fisher Scientific). Slides were baked for 1 hour at 60°C, deparaffinized in xylene, and dehydrated in 100% ethanol. Sections were treated with H_2_O_2_ (ACD, 32281) for 10 minutes at room temperature and then heated to mild boil (98°C–102°C) in 1× target retrieval reagent buffer (ACD) for 15 minutes. Protease Plus (ACD, 32281) was applied to sections for 30 minutes at 40°C in a HybEZ Oven (ACD). Hybridization with target probes, preamplifier, amplifier, fluorescent labels, and wash buffer (ACD) was done according to manufacture’ instructions for Multiplex Fluorescent Reagent Kit version 2 (catalog 322809). Parallel sections were incubated with ACD positive and negative control probes. Sections were covered with ProLong Gold Antifade Mounting (Invitrogen). Mouse probes used were: *Wnt2*-C1 (catalog 323601-C1), *Wnt5a*-C1 (catalog 316791-C1), *Wnt5a*-C2 (catalog 316791-C2), *Wnt5a*-C3 (catalog 316791-C3), *PDGFRa-*C2 (480661-C2), *Sftpc*-C3 (catalog 314101-C3), *Axin2*-C1 (catalog 400331-C1), mouse negative control (catalog 320871), and mouse positive control (catalog 320881), from ACD. Opal fluorophores (Opal 520, FP1487001KT; Opal 620, FP1495001KT; Opal 690, FP1497001KT) from Akoya (PerkinElmer) were used at 1:1,500 dilution in Multiplex TSA buffer (ACD, 322809). Images were captured on a Nikon A1R confocal microscope with 20***×*** or 40***×*** original magnification, followed by uniform processing and pseudo-coloring in Fiji (https://imagej.net/Fiji).

For cytospins, freshly isolated mouse lung PDGFRα^+^ fibroblasts were centrifuged at 300*g* for 10 minutes at 4^o^C and resuspended in PBS. Multiple replicates of 20,000 cells in 150 μL volume were loaded to Cytofunnels (Shandon) and applied to Superfrost Plus charged slides (Thermo Fisher Scientific) by centrifugation in Cytospin 3 centrifuge (Thermo Fisher Scientific) at 113*g* for 5 minutes at room temperature. Adherent cells were fixed, dehydrated, stored, rehydrated, and pretreated with hydrogen peroxide (ACD, 322381) and protease III (ACD, 322340) according to sample preparation method (ACD RNAscope Multiplex Fluorescent v2 Assay) for cultured adherent cells.

### Spatial analysis of the mouse lung.

To assess spatial proximity of *Wnt2*, *Wnt5a*, and *Pdgfra* mRNA signals to *Sftpc*^+^ AT2 cells, quantification was performed using Nikon NIS-Elements software version 5.30.02 using the General Analysis module, NearstObjDist algorithm. For each RNA-FISH probe, thresholding allowed us to distinguish specific signal over background, generating a binary layer based on the size and intensity of objects (a single probe detected by NIS-Element algorithm) using Bright Spot Detection thresholding (specifically the *Bright*, *Clustered* detection method). General diameter and contrast were then adjusted for each object and were then converted to “spots” to produce a 3-pixel, centroid of objects. This converted the RNA-FISH signal into objects (circle/spot) based on signal density, so distances could be measured between spot centers. The smallest distance a given “spot” of channel/color (e.g., *Pdgfra*^+^ spot) was from the other 2 channel/color “spots” (e.g., *Sftpc*^+^ spot or *Wnt2*^+^ spot) was measured for all image spots. Mean shortest distance measurements were quantified and plotted in Prism.

### Identification of subtypes of Pdgfra^+^ fibroblast following isolation and cytospin.

Following FISH protocol, mouse probes for *Pdgfra*, *Wnt2*, and *Wnt5a* were used to distinguish subtypes of *Pdgfra* fibroblasts. Signals of *Pdgfra*^+^ and *Wnts* were captured by Bright Spot Detection thresholding using Nikon NIS-Elements, with similar methodology as discussed previously. *Pdgfra*^+^ objects were then dilated by 5 μm and combined with DAPI signal to form new binaries — a representation of *Pdgfra*^+^ fibroblasts. To capture plausible RNA signals within “*Pdgfra^+^* fibroblasts,” simple Boolean operator expressions (using the Intersect function of General Analysis, which allows users to combine multiple binary layers into a single layer for further analysis) were used to identify subpopulations of *Pdgfra*^+^ cells overlapping with *Wnt* binary layers, which were also dilated by 4 μm for detection.

### βCat/TCF reporter assay.

A549 cells (1.5 ***×*** 10^5^ cell/well) were transfected with Super8-TOPflash (containing 8 TCF-consensus binding sites) or 1 μg of Super8-FOPflash (8 mutant TCF-binding sites) reporter plasmids (provided by Randall Moon, University of Washington, Seattle, Washington, USA) using Lipofectamine 2000 (Invitrogen); a thymidine kinase-Renilla plasmid (0.1 μg) was also included to normalize luciferase values to the efficiency of transfection as previously described (17). After 4 hours of transfection, media were replaced, and cells were incubated overnight under normocapnic or hypercapnic conditions. The next morning, rWnt3 (50ng/mL) or rWnt5a (50ng/mL) was added to the media, and cells were incubated for another 24 hours. At the end of the incubation, A549 cells were solubilized using the Dual-Luciferase Assay Kit (Promega), and luciferase activity was quantified using a microplate dual-injector luminometer (Veritas) according to manufacturer’s instructions. Briefly, cells in each well of a 6-well plate were incubated on ice with 250 μL of a 1***×*** passive lysis buffer for 15 minutes followed by scraping to lift adherent cells. In total, 20 μL of cell lysate was mixed with 100 μL of supplied Luciferase Assay Reagent II, and firefly luciferase was measured.

### Measurement of βcat signaling pool.

AT2 cells were isolated from rat lungs as described (17, 65). In total, 3 ***×*** 10^6^ AT2 cells were plated to 100 mm (low density), 60 mm (medium density), and 35 mm (high density) culture dishes and immediately infected with 20 pfu/cell adenovirus (Vector Biolabs, 1 ***×*** 10^10^ to 1 ***×*** 10^12^ pfu/mL): Ad-CMV-GFP, Ad-Wnt3a, and Ad-Wnt5a. Infection efficiency of (>90%) was confirmed by visualizing GFP expression in living cells. Cells were lysed after 72 hours as described (17). Protein lysates were processed for GST-ICAT pulldown as described (43). Briefly, 100 μg of lysate was saved as input fraction. In total, 1000 μg of lysate was combined with 50 μL of a 50:50 slurry of prewashed glutathione-sepharose beads (GE, 4B) and 20 μg of GST-ICAT and were rocked at 4°C for 2 hours. Samples were centrifuged at 12,000*g* for 1 minute to pellet beads, and a 5 μL aliquot of supernatant was collected as the unbound fraction. Beads were washed with 0.1% triton buffer and centrifuged 3 times as described above, and the final pellet resuspended in 35 μL 2***×*** SDS loading buffer as bound pulldown fraction. Protein lysates were run on 6% acrylamide gel and transferred to nitrocellulose for immunoblotting as described above. Primary antibodies included the following: mouse anti-ABC (clone 8E7, MilliporeSigma, 05-665; lot 2275597; 1:1,000), mouse anti–βcat (clone 14, BD Biosciences, 610154; lot 1085870; 1:1,000 or 1:5,000), mouse anti–E-cadherin (clone 36, BD Biosciences, 610182; lot 4353669; 1:1,000), and rabbit anti-GAPDH (Santa Cruz Biotechnology Inc., sc-25778; lot D1613; 1:1,000).

### Data and materials availability.

The RNA-Seq data sets are available at the NCBI’s Gene Expression Omnibus (GEO) database, accession nos. GSE139426 and GSE164733.

### Statistics.

Analyses of significance were performed using GraphPad Prism (v.8.4.2) software. A *P* ≤ 0.05 was considered statistically significant. A standard 2-tailed unpaired Student’s *t* test was used for statistical analysis of 2 groups. One-way ANOVA, followed by analysis-specific post hoc tests, was carried out when more than 2 variables were compared. Unless stated otherwise, data are presented mean ± SD overlaid with individual data points representing replicates.

### Study approval.

Animal work was conducted in accordance with the recommendations in the *Guide for the Care and Use of Laboratory Animals* (National Academies Press, 2011). All procedures were approved by the Northwestern University’s IACUC (no. IS00010662). All strains, including WT mice, were bred and housed at a barrier- and pathogen-free facility at the Center for Comparative Medicine at Northwestern University.

## Author contributions

LAD, LCW, CJG, and JIS conceived and designed the research. LAD, LCW, NDM, HH, PLB, DC, ASF, AW, MMH, HAV, MS, SMCM, VK, IV, and CER performed and analyzed the experiments. LAD, LCW, NDM, HH, PLB, AVM, and CJG analyzed the experimental data. ZR and AVM performed the bioinformatic analysis. LAD, GRSB, CJG, and JIS wrote the manuscript. All authors provided edits and feedback on the manuscript.

## Supplementary Material

Supplemental data

## Version 1 01/10/2023

In-Press Preview

## Version 2 02/22/2023

Electronic publication
