# Supplementary material for: Hypercapnia alters stroma-derived Wnt production to limit β-catenin signaling and proliferation in AT2 cells
Source: JCI Insight. 2023 Feb 22;8(4):e159331. doi: 10.1172/jci.insight.159331 (PMC9977495; doi:10.1172/jci.insight.159331)
Supplement: Supplemental data [file jciinsight-8-159331-s244.pdf]

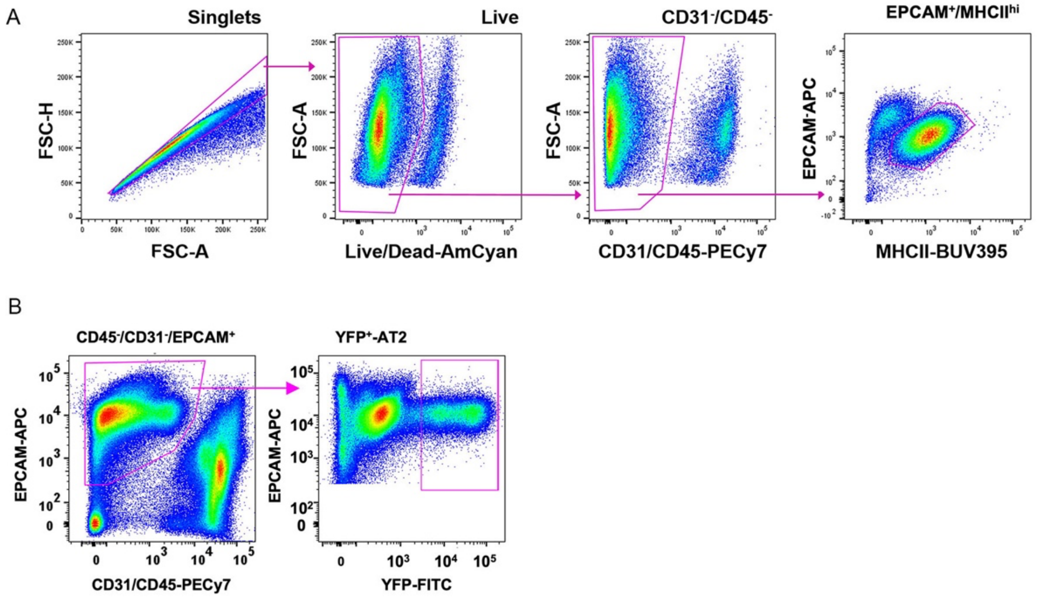

Supplemental Figure 1. Gating strategies used to identify AT2 cells in lung single cell populations. (A) Representative flow cytometry plots from a wild-type adult mouse and (B) Tamoxifen-treated $Sftp_{CreERT2}:R26R_{eYFP}$.

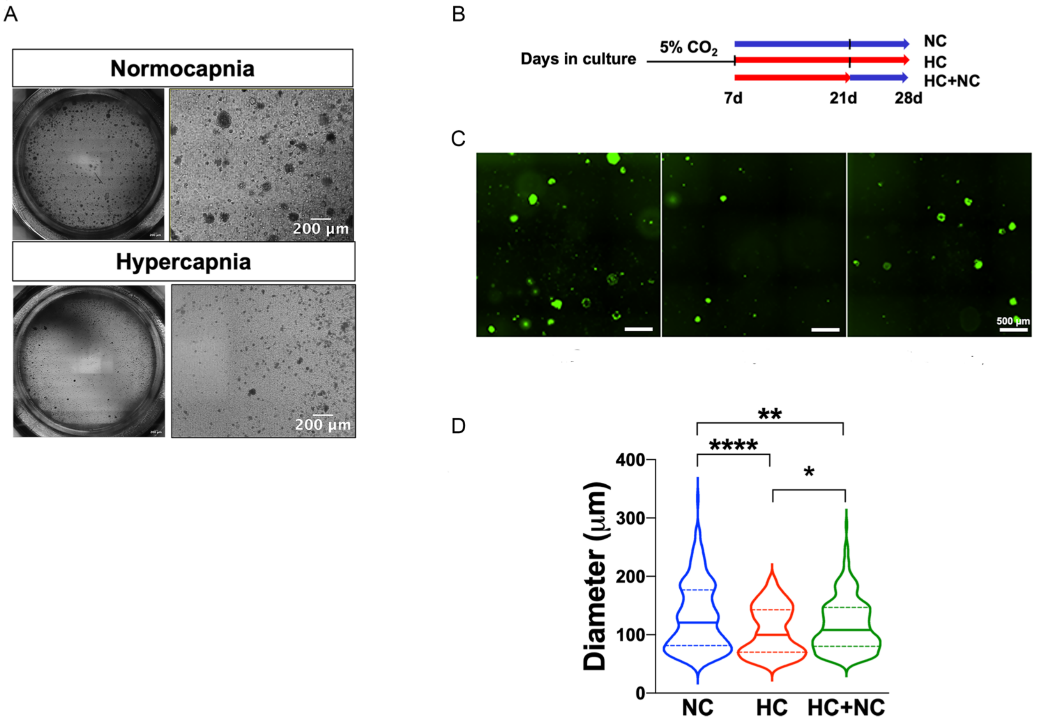

Supplemental Figure 2. Hypercapnia limits AT2 cell proliferation in 3D culture organoids. (**A**) Representative images of 21 days organoid cultures of wild-type AT2 cells in normocapnia or hypercapnia. Scale bars: 200µm. (**B**) Schematic of experiments designed to co-culture AT2 isolated from *Sftp*^{CreERT2}*:R26R*^{EYFP} AT2 and wild-type mesenchymal cells in organoid assays, depicting the switch to normocapnia (5% CO_2; NC) or hypercapnia (20% CO_2; HC) media. (**C**) Representative images of organoid cultures in normocapnia or hypercapnia. Scale bars: 500µm. (**D**) Graph depicts the inhibitory effect of hypercapnia on organoid size and the rescue after switching the media back to normocapnia. Median with interquartile range, n=6. ANOVA plus Sidak's multiple comparisons test and *P<0.05; ** P<0.01, ****P<0.0001.

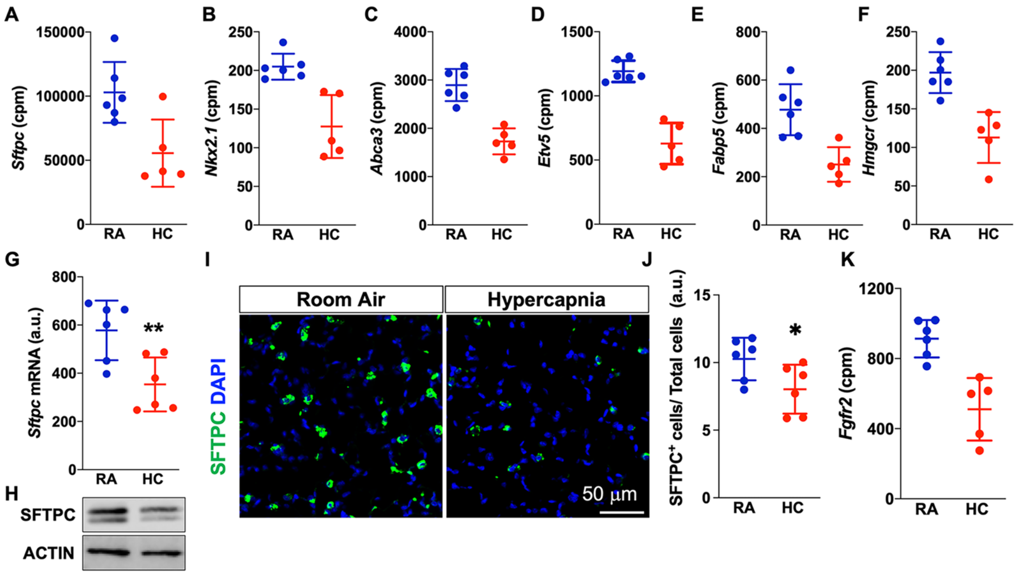

Supplemental Figure 3. Transcriptomic analysis of AT2 cells shows that hypercapnia decreases the expression of AT2 markers. (A-F) Bulk RNASeq was performed on flow cytometry sorted AT2 cells cell sorting from lung tissue from mice kept breathing room air (RA, n=6) or exposed to 10% CO_2 (HC, n=5). Hypercapnia decreases the expression of AT2 markers. Expression of selected DEG markers of AT2 cell regulated by hypercapnia. **(G)** Quantification of *Sftpc* mRNA in AT2 cells isolated from mice exposed to 10% CO2. **(H)** A representative SFTPC Western blot with specific antibodies is shown. ACTIN was used as a loading control. **(I and J)** Quantification of SFTPC positive cells in the alveolar region of adult mouse lung from mice kept breathing RA or exposed to HC for 21 days. n=5.Scale bar 50µm **(K)** Hypercapnia decreases the expression of *Fgfr2*. Adjusted p values from RNA-Seq analysis are shown. Data are mean ± SD. **p*<0.05; ***p*<0.01

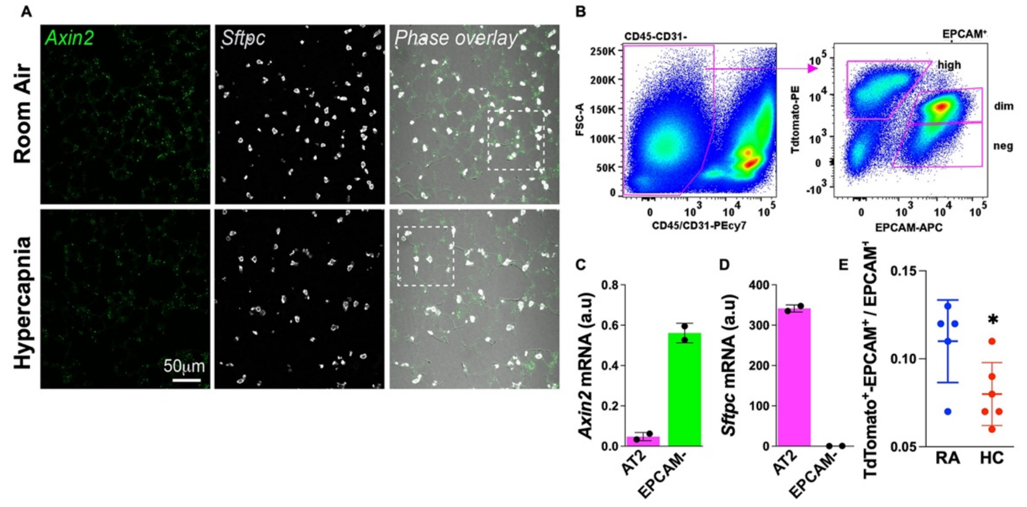

Supplemental Figure 4. Hypercapnia decreases Wnt/βcat signaling in AT2 cells. (**A**) Low magnification of *in situ* RNA hybridization showing the decreased number of *Axin2*$^+$-AT2 cells in mice exposed to hypercapnia shown in Figure 3B. Scale bar: 50 μm. **(B)** Gating strategy used to isolate TdTom$^+$ -AT2 cells from *Axin2*$^{CreERT2-TdTom}$ mice. (**C-D**) Expression of *Axin2* and *Sftpc* mRNA by RT-qPCR in AT2 and EPCAM$^-$ cells isolated from *Axin2*$^{CreERT2-TdTom}$ mice. (**E**) Number of TdTom$^+$-AT2 cells from *Axin2*$^{CreERT2-TdTom}$ was determined by flow cytometry. n=10 mice, graph shows the data from two of three independent experiments. Data are mean ± SD.* Student's *t*-test. $^*P<0.05$.

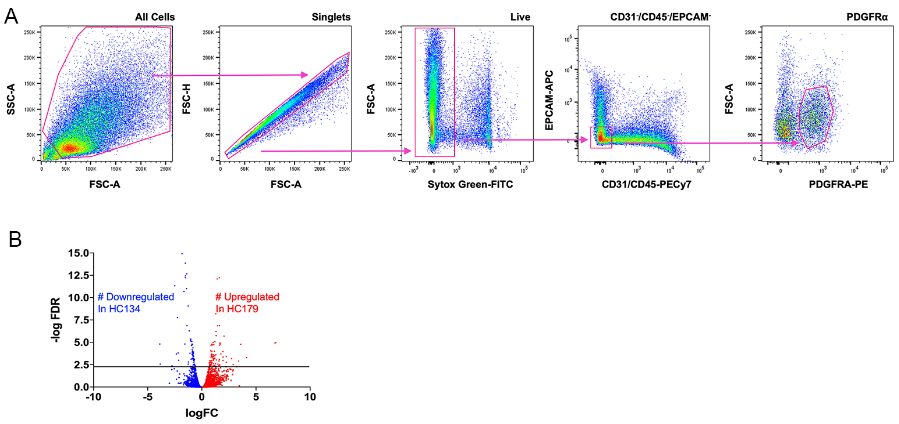

Supplementary Figure 5. Hypercapnia alters Wnt expression in PDGFRα⁺ fibroblasts. (A) Gating strategy to isolate PDGFRα⁺ from mice. **(B)** Volcano plot of the expression of Wnt genes in PDGFRα⁺ fibroblasts. FDR values from RNA-Seq analysis are shown.

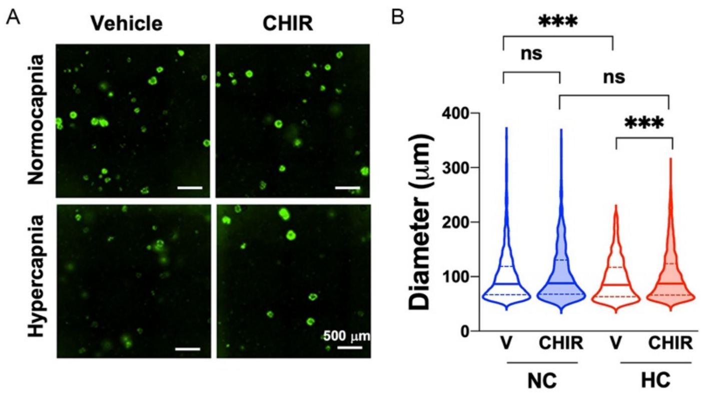

Supplemental Figure 6. Activation of βcat signaling rescues AT2-proliferative capacity during hypercapnia. **(A-B)** Representative fluorescent images of typical day 21 3D-cultures of AT2 isolated from $Sftp^{CreERT2}$:$R26R^{eYFP}$ mice. Organoids were cultured in normocapnia or hypercapnia with or without the addition of CHIR (20 nM) as described in Figure 1. Graphs depict the effect CHIR treatment on normocapnia and hypercapnia organoid size n=6 mice in 3 independent experiments. Scale bars: 500 μm.

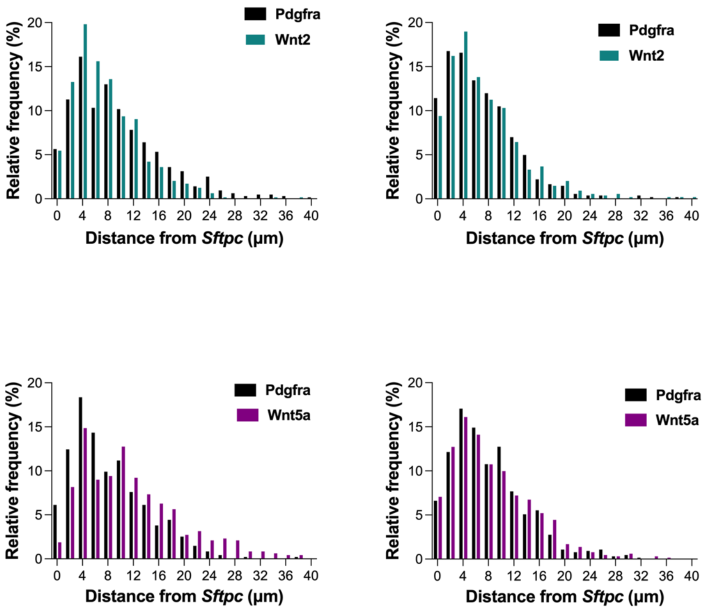

Supplemental Figure 7. Percentage of the shortest mean distance to *Sftpc*. Representative histograms depicting relative frequency expressed as percentage for 3 fields of vision per mouse for the data in Figure 7.

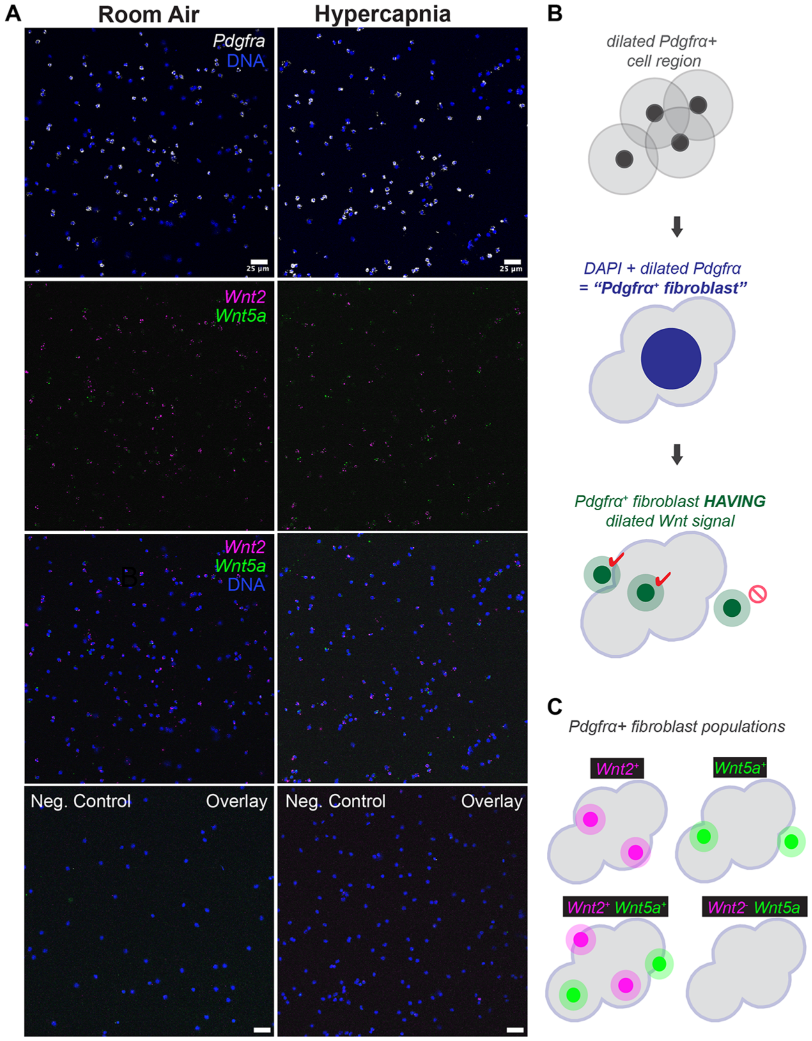

Supplemental Figure 8. **Hypercapnia alters distribution of *Wnt2/Wnt5a* expression in PDGFRα$^+$fibroblasts using cytospin analysis**. (**A**) Confocal mages of PDGFRα$^+$-flow sorted fibroblasts subjected to RNA-FISH with probes to *Pdgfra* (white), *Wnt2* (magenta) and *Wnt5a* (green). Negative control also shown. Hoechst/DNA stain in blue. Scale bar = 25 μm. (**B)** Schematic of method to quantify co-occurrence of *Wnt2* and *Wnt5a* signals within *Pdgfra*-regions. **C**. Schematic of outcomes observed. Graphical quantification in Figure 8. Scale bars: 25 μm

Supplemental Table 1.

Nucleotide sequence of primers used for qRT-PCR

Gene	Forward Primer	Reverse Primer
Actin	5'-CAT CCG TAA AGA CCT CTA TGC CAA C-3'	5'-ATG GAG CCA CCG ATC CAC A-3'
Axin2	5'-ACT GGG TCG CTT CTC TTG AA-3'	5'-CTC CCC ACC TTG AAT GAA GA-3'
Sftpc	5'-AGC AAA GAG GTC CTG ATG GA-3'	5'-GAG CAG AGC CCC TAC AAT CAA-3'
Wnt4	5'-CGA GCA ATT GGC TGT ACC TG-3'	5'-CCT CAA GGT TCC GTT TGC AC-3'
Wnt5a	5'-CTG GCA GGA CTT TCT CAA GG-3'	5'-GTC TCT CGG CTG CCT ATT TC-3'
Wnt11	5'-ACC TGC TTG ACC TGG AGA GA-3'	5'-AGC CCG TAG CTG AGG TTG T-3'
Gapdh	5’-AAC TTT GGC ATT GTG GAA GG-3’	5’-ACA CAT TGG GGG TAG GAA CA-3’
